# Design, synthesis, molecular modelling and antitumor evaluation of *S*-glucosylated rhodanines through topo II inhibition and DNA intercalation

**DOI:** 10.1080/14756366.2022.2163996

**Published:** 2023-01-11

**Authors:** Ahmed I. Khodair, Fatimah M. Alzahrani, Mohamed K. Awad, Siham A. Al-Issa, Ghaferah H. Al-Hazmi, Mohamed S. Nafie

**Affiliations:** a Chemistry Department, Faculty of Science, Kafrelsheikh University, Kafrelsheikh, Egypt; b Department of Chemistry, College of Science, Princess Nourah bint Abdulrahman University, Riyadh, Saudi Arabia; c Theoretical Applied Chemistry Unit (TACU), Chemistry Department, Faculty of Science, Tanta University, Tanta, Egypt; d Chemistry Department, Faculty of Science, Suez Canal University, Ismailia, Egypt

**Keywords:** Thiazolidinones, antitumor activity, apoptosis, molecular modelling, DFT calculations

## Abstract

In the present study, 5-arylidene rhodanine derivatives **3a–f**, *N*-glucosylation rhodanine **6**, *S*-glucosylation rhodanine **7**, *N*-glucoside rhodanine **8** and *S*-glucosylation 5-arylidene rhodanines **13a–c** were synthesised and screened for cytotoxicity against a panel of cancer cells with investigating the effective molecular target and mechanistic cell death. The anomers were separated by flash column chromatography and their configurations were assigned by NMR spectroscopy. The stable structures of the compounds under study were modelled on a molecular level, and DFT calculations were carried out at the B3LYP/6-31 + G (d,p) level to examine their electronic and geometric features. A good correlation between the quantum chemical descriptors and experimental observations was found. Interestingly, compound **6** induced potent cytotoxicity against MCF-7, HepG2 and A549 cells, with IC_50_ values of 11.7, 0.21, and 1.7 µM, compared to Dox 7.67, 8.28, and 6.62 µM, respectively. For the molecular target, compound **6** exhibited topoisomerase II inhibition and DNA intercalation with IC_50_ values of 6.9 and 19.6 µM, respectively compared to Dox (IC_50_ = 9.65 and 31.27 µM). Additionally, compound **6** treatmnet significantly activated apoptotic cell death in HepG2 cells by 80.7-fold, it induced total apoptosis by 34.73% (23.07% for early apoptosis, 11.66% for late apoptosis) compared to the untreated control group (0.43%) arresting the cell population at the *S*-phase by 49.6% compared to control 39.15%. Finally, compound **6** upregulated the apoptosis-related genes, while it inhibted the Bcl-2 expression. Hence, glucosylated rhodanines may serve as a promising drug candidates against cancer with promising topoisomerase II and DNA intercalation.

## Graphical Abstract

**Figure:**
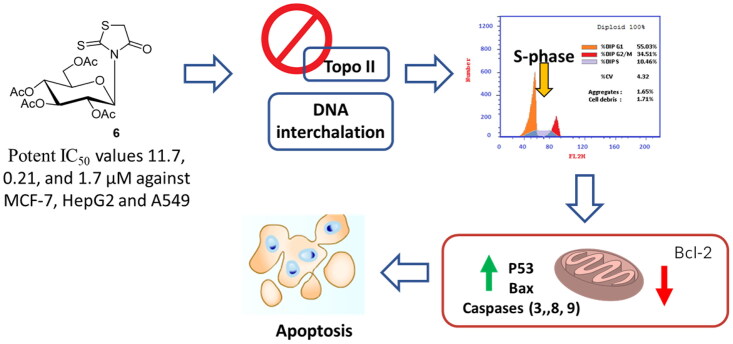

## Introduction

Worldwide, cancer is a major contributor to mortality rates and a major bottleneck to extending the human lifespan. Estimates for 2020 indicate that there would be 19.3 million new cases of cancer and about 10.0 million deaths from cancer worldwide. With an expected 1.8 million fatalities (18%), lung cancer continued to be the largest cause of cancer-related mortality. This was followed by colorectal (9.4%), liver (8.3%), stomach (7.5%), and female breast (6.9%) cancers^1^. With the continuous failure of current medicines on one side and the development of drug resistance on the other, cancer offers one of the greatest challenges to medical science^2^. Topoisomerases are nuclear enzymes that temporarily break DNA strands to facilitate DNA topological manipulation^3^^,^^4^. Topoisomerase I intentionally tear off individual molecules. In order to unwind tightly wound DNA, topoisomerase II creates double-strand breaks and shuttles the strands through a nick in the strand^5^. Topoisomerases are enzymes that are present in all eukaryotic cells and are crucial to their survival^6^. One of the most effective ways to combat cancer is by inhibiting human DNA topoisomerase II. Drugs that inhibit topoisomerase II have shown great promise in the clinic, however, drug resistance in cancer cells can reduce their usefulness^7^. Combination therapy and multitarget medications have been proposed in numerous research to increase the efficacy of anticancer medication^8^. Inhibitors of topoisomerase have been used extensively in chemotherapy. Etoposide, doxorubicin, daunorubicin, mitoxantrone, teniposide, and amsacrine are some of the most widely utilised topoisomerase II inhibitors^9^. Topo II inhibitors were initially quite effective in treating the disease, but further treatments proved unsuccessful^10^. As a result, new medications targeting topoisomerase II are needed to eliminate these limitations^11^.

2-Thioxo-4-thiazolidinone and its derivatives have been the subject of organic synthesis research for well over half a century due to their usefulness in a wide variety of chemical and biological processes. From a biological point of view, derivatives of 2-thioxo-4-thiazolidinone have been demonstrated to possess biological activities, including antibacterial^12–15^, antifungal^16^^,^^17^, anticonvulsant^18^, anticancer^19^, antihuman immunodeficiency virus type 1 (HIV-1)^20–22^, antimicrobial^23^, antidiabetic^24^, antituberculotic^25–27^, antiparasitic^28^, hypnotic^29^ and anthelmintic activities^30^. Nucleic acid synthesis also involves the use of 2-thioxo-4-thiazolidinones, which function as analogues of purine bases^31^. Over the past decade, thiazole nucleoside analogues have emerged as powerful and increasingly essential antimetabolite agents^32^^,^^33^. *N*-(beta-D-glucopyranosyl)-5-(4-nitrobenzylidene)-2-thioxo-4-thiazolidinone demonstrated antiviral efficacy by inhibiting viral RNA synthesis, and although *N*-glucosyl derivatives of 2-thioxo-4-thiazolidinones are not widely studied, they have shown promise as antiviral agents^34^. Previous studies of glycosyl derivatives of structurally related heterocyclic systems were reported^35–50^. While bearing in mind the biological relevance of 2-thioxo-4-thiazolidinones, we have continued our work on the synthesis of new nucleosides as possible antiviral and anticancer medicines^51–58^. Herein, we detail the design, synthesis, anticancer screening, and spectroscopy of a panel of *N*- and *S*-glucosylated analogues with thioxo-4-thiazolidinone bases. New synthesis methods have enabled the first preparation of *N*- and *S*-glucosyl derivatives of 2-thioxo-4-thiazolidinones. Metwally et al.^24^ were able to synthesise *N*-glycosyl derivatives of 2-thioxo-4-thiazolidinones without resorting to the *S*-glycosylation of 2-thioxo-4-thiazolidinone. Conversely, they were unable to synthesise 2-thioxo-4-thiazolidine *N*-glycosyl derivatives that would have been required for this process. Based on the results of these analyses, we investigated alternative synthetic routes leading to 2-thioxo-4-thiazolidinone nucleosides for use as antiviral and anticancer medicines. Nitrogen glycosylated compounds and their sulphur counterparts with 2-thioxo-4-thiazolidinone bases are synthesised and analysed for conformational stability and anticancer activity in this study. To the best of our knowledge, this represents the first time that *S*-glycosides of 2-thioxo-4-thiazolidinone have been prepared using novel synthetic approaches.

Computational chemistry has come a long way in the past few decades, and it is now commonly used alongside experimental methods to study organic and biological structures and reactions. Structures, characteristics of molecules, processes, and selectivity of reactions can all be better understood with the use of computations^59^. Density functional theory (DFT) is widely used to calculate many different types of molecular properties, including but not limited to molecular structures, vibrational frequencies, chemical shifts, non-linear optical (NLO) effects, natural bond orbital (NBO) analysis, molecular electrostatic potential, frontier molecular orbitals, and thermodynamic properties^60–68^. Herein, we detail the design, synthesis, anticancer screening, and spectroscopic analysis of a series of nitrogen glucosylated carrying 2-thioxo-4-thiazolidinone bases. The purpose of this work is to use density functional theory to analyse how alterations to molecular and electronic structure affect the biological activity of the substances under research, and to try to locate a strong correlation between theoretical data and actual observations.

## Results and discussion

### Chemistry

**Scheme 1. Synthesis of 5-((Z)-arylidene)-2-thioxo-4-thiazolidinone derivatives (3a–f).**
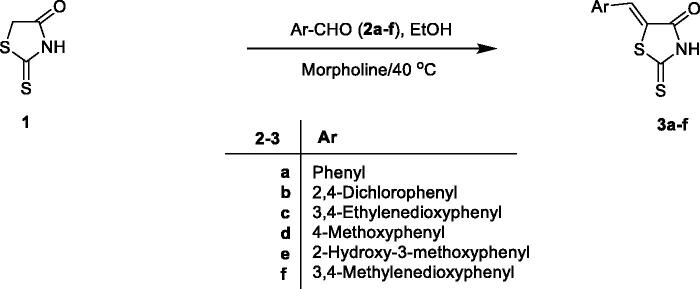


2-Thioxo-4-thiazolidinone (**1**) was reacted with aromatic aldehydes (**2a–f**) in ethanol and morpholine as secondary amine at room temperature to give 5-((*Z*)-arylidene)-2-thioxo-4-thiazolidinones (**3a–f**) (Scheme 1)^69–71^. The compositions of 5-((*Z*)-arylidene)-2-thioxo-4-thiazolidinones (**3a–f**) were confirmed on the basis of elemental analysis and spectroscopic data (IR, ^1^H NMR, ^13^C NMR and MS). The infra-red absorption spectrum of compound **3a** was characterised by the presence of signals for the NH and CO groups at 3190 and 1726 cm^−1^, respectively. The ^1^H NMR spectrum (DMSO-d_6_) of 5-((*Z*)-benzylidene)-2-thioxo-4-thiazolidinone (**3a**) showed a single piece at δ 7.62 ppm assigned to the vinyl proton, indicating a *Z* configuration of the outer cyclic double bond. This is consistent with the ^1^H NMR spectrum (DMSO-d_6_) of 5-((Z)-(2-pyridylmethylene)-2-thioxo-4-thiazolidinone showing its vinyl proton at δ 7.70 ppm^72^. The ^13^C NMR spectrum (DMSO-d_6_) for 5-((*Z*)-benzylidene)-2-thioxo-4-thiazolidinone (**3a**) showed signals at δ 125.90, 169.76 and 196.70 ppm assigned to vinyl, carbonyl and thiocarbonyl carbons, indicating respectively the presence of a *Z* configuration of the outer cyclic double bond. This is in agreement with the ^13^C NMR spectrum (DMSO-d_6_) of 5-((*Z*)-(2-pyridylmethylene)-2-thioxo-4-thiazolidinone showing vinyl, carbonyl and thiocarbonyl carbons respectively at δ 124.66, 170.05 and 202.66 ppm^72^ (Scheme 1).

**Scheme 2. Synthesis of 3-(4-morpholinomethyl)-2-thioxo-4-thiazolidinone (10) and 2-methylmercapto-4-thiazolidinone (11).**
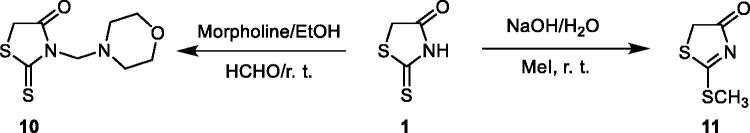


**Scheme 3. Synthesis of 2-thioxo-4-thiazolidinone nucleosides derivatives 6–8.**
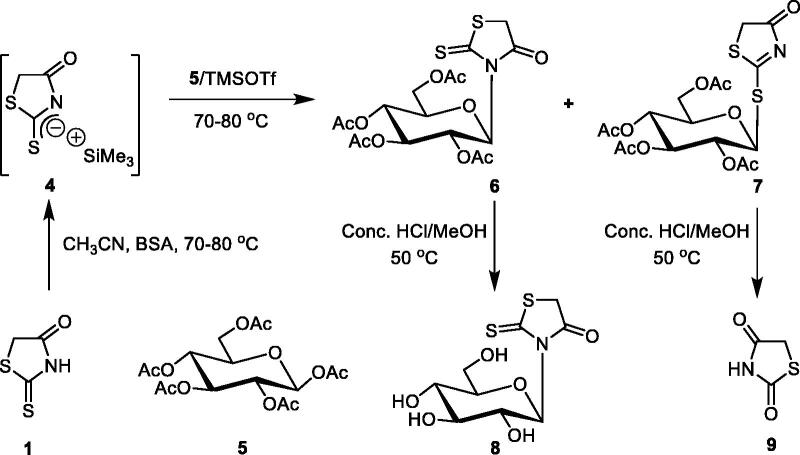


The silylation of the nucleoside base **1** was accomplished with bis(trimethylsilyl)acetamide (BSA) in anhydrous MeCN at 70–80 °C, and furnished the trimethylsilylated derivative **4**. This derivative **4** was condensed, devised by Vorbrüggen et al.^73^, with 1,2,3,4,6-penta-*O*-acetyl-α-D-glucopyranose (**5**) in the presence of trimethylsilyl trifluoromethyanesulfonate (TMSOTf) as catalyst at 70–80 °C for 60 min. The nucleosides **6** and **7** were isolated by silica gel column chromatography in 6% and 30% yields, respectively. The structures of **6** and **7** were established and confirmed by elemental analyses and spectral data (IR, ^1^H NMR, ^13^C NMR and MS). The absence of signal for NH and the presence of signal for the thiocarbonyl group at *v_max_* 1225 cm^−1^ characterised the IR absorption spectrum of compound **6**. While the IR absorption spectrum of compound **7** was characterised by the absence of signal for thiocarbonyl group. The ^1^H NMR spectrum of compound **6** showed the anomeric proton as a doublet at δ_H_ 6.82 ppm (*J* = 9.30 Hz) indicating the presence of the β-D-glucopyranose moiety^35–39^. The ^13^C NMR (CDCl_3_) spectrum of compound **6** showed a singlet at δ_C_ 172.1 and 201.5 ppm assigned to the carbonyl at C-4 and the thiocarbonyl group at C-2, respectively. These data are also in agreement with the ^13^C NMR (CDCl_3_) spectrum of 3-(4-morpholinomethyl)-2-thioxo-4-thiazolidinone (**10**) (Scheme 2). The later was prepared from the reaction of **1** with morpholine and formaldehyde in EtOH at room temperature, since the carbonyl at C-4 appears at δ_C_ 175.1 ppm and the thiocarbonyl group at C-2 appears at δ_C_ 203.2 ppm indicating the presence of *N*-glycosylation. The ^1^H NMR spectrum of compound **7** showed the anomeric proton as a doublet at δ_H_ 5.82 ppm (*J* = 10.4 Hz) indicating the presence of the β-D-glucopyranose moiety^35–39^. The ^13^C NMR (CDCl_3_) spectrum of compound **7** showed a singlet at δ_c_ 186.5 and 198.83 ppm assigned to the carbonyl at C-4 and the thiocarbonyl group at C-2, respectively. These data are also in agreement with the ^13^C NMR (CDCl_3_) spectrum of hitherto known 2-methylmercapto-4-thiazolidinone (**11**)^70^ (Scheme 2) since the methylmercapto at C-2 appears at δ_C_ 15.9 ppm, the carbonyl at C-4 appears at δ_C_ 187.1 ppm and the thiocarbonyl group at C-2 appears at δ_C_ 202.5 ppm indicating the presence of *S*-glycosylation. Treatment of the protected nucleoside **6** with conc. HCl/MeOH (3.5%) at 50 °C for 2 h afforded in both cases the corresponding deprotected nucleoside **8** indicating *N*-glycosylation. In the same manner, treatment of **7** with conc. HCl/MeOH (3.5%) at 50 °C for 2 h furnished hitherto known 2,4-thazolidinedione (**9**)^74^ indicating *S*-glycosylation. This type of cleavage explains why we did not successful in the preparation of the corresponding deprotected nucleoside of **7** (Scheme 3).

**Scheme 4. Synthesis of 5-((Z)-arylidene-2-(2′,3′,4′,6′-tetra-O-acetyl-β-D-glucopyranosyl)-2-thioxo-4-thiazolidinones derivatives (13a–c).**
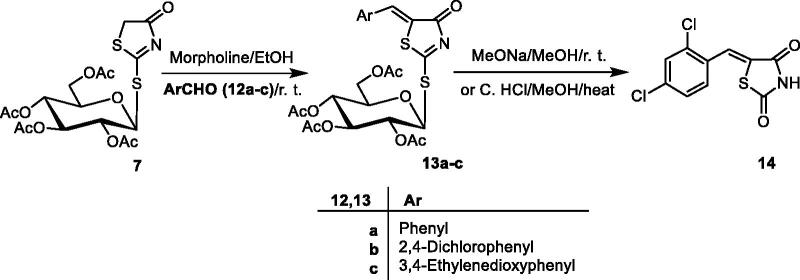


Correspondingly, the protected nucleoside **7** was condensed with the appropriate aromatic aldehydes (**12a–c**) in ethanol in the presence of morpholine as secondary amine catalyst to afford the corresponding 5-((*Z*)-arylidene-2-(2′,3′,4′,6′-tetra-*O*-acetyl-β-D-glucopyranosyl)-2-thioxo-4-thiazolidinones (**13a–c**) in good yields. Treatment of the protected nucleoside **13b** with a solution of sodium methoxide in methanol furnished 5-((*Z*)-2,4-dichlorobenzylidene)-2,4-thiazolidindione (**14**). This type of cleavage explains why we did not successful in the preparation of the corresponding deprotected nucleosides of **13a–c**. The structures of **13a–c** and **14** were established and confirmed by elemental analyses and spectral data (IR, ^1^H NMR, ^13^C NMR and MS). The absence of signals for the NH and CS groups characterises the IR absorption spectra of **13a**. The ^1^H NMR (CDCl_3_) spectrum of 5-((*Z*)-benzylidene-2-(2′,3′,4′,6′-tetra-*O*-acetyl-β-D-glucopyranosyl)-2-thioxo-4-thiazolidinones (**13a**) showed a single piece at δ_H_ 7.91 ppm assigned to the vinyl proton, indicating a *Z* configuration of the cyclic double bond. This is consistent with the ^1^H NMR (CDCl_3_) spectrum of 5-((*Z*)-benzylidene)-2-allylmercapto-4-thiazolidinone showing a vinyl proton at δ H 7.84 ppm^70^. While the abnormal proton appears as a doublet at δ H 5.98 ppm (*J* = 10.4 Hz), indicating the presence of the β-D-glucopyranose moiety^35–39^. It showed a ^13^C NMR spectrum (CDCl_3_) for 5-((*Z*)-benzylidene-2-(2′,3′,4′,6′-tetra-*O*-acetyl-β-D-glucopyranosyl)-2-thioxo-4-thiazolidinones (**13a**) singlet at C 178.8 and 189.2 ppm assigned to a carbonyl at C-4 and a CS group at C-2, respectively. These data are also consistent with the ^13^C NMR (CDCl_3_) spectrum of 5-((*Z*)-benzylidene)-2-allylmercapto-4-thiazolidinone^70^, since the carbonyl at C-4 appears at 179.8 ppm. The CS group is shown in C-2 at −191.8 ppm, indicating a binding to *S*-glycosylation (Scheme 4).

## Computational method

DFT (density functional theory) was utilised to optimise the molecule structures of the substances under study by utilising the Beck’s three parameter exchange functional and the Lee-Yang-Parr non local correlation functional (B3LYP)^75–77^ with 6–31 + G(d,p) basis set which is implemented in Gaussian 09 program package^78^. The DFT/B3LYP combination was used to determine molecular characteristics relevant to molecular reactivity^79^. Highest occupied molecular orbital (HOMO), lowest unoccupied molecular orbital (LUMO), global hardness and softness, electronegativity, electron affinity, and ionisation potential are examples of molecular characteristics^80–82^.

## Quantum chemical study

Quantum chemistry approaches and molecular modelling techniques can describe a wide variety of molecular characteristics that characterise the reactivity, shape, and binding properties of an entire molecule, a molecule fragment, or a substituent. Some substances’ biological activity was shown to be affected by changing structural factors, and these changes were explored using quantum chemical simulations. According to the results of the computations, the geometrical structures of the organic molecules under study are not planar.

Our calculations began with a comparison of the Z- and E-form stabilities of the compounds under study; we found that, for all molecules under study, the Z-form is more stable than the E-form by a factor of 0.003–0.062 au, which is in good agreement with experimental results. Thus, we used density functional theory (DFT) computations to determine the Z-form stable structures of all molecules.

We started our calculations to make a comparison between the stability of the investigated compounds in *Z*- and *E*-forms and the calculations showed that they are more stable in the *Z*-form than *E*-form by 0.003–0.062 au, for all investigated molecules which is in a good agreement with the experimental observations. So, we performed DFT calculations on the stable structures of Z-form for all molecules.

### Compounds 3a–f, 10 and 14

The optimised molecular structures with minimum energies obtained from the calculations of the investigated compounds are shown in Figure 1. The calculations were done to investigate the effect of substituents on the biological activity of the thioxothiazoline-4-one **3a** compound. In general, it was shown from the quantum chemical parameters obtained from the calculations that the presence of substituents on thioxothiazoline-4-one increase the reactivity of the inhibitors **3b–f**.

Figure 1.The optimised molecular structures of the investigated inhibitors obtained from the calculations.

**Figure:**
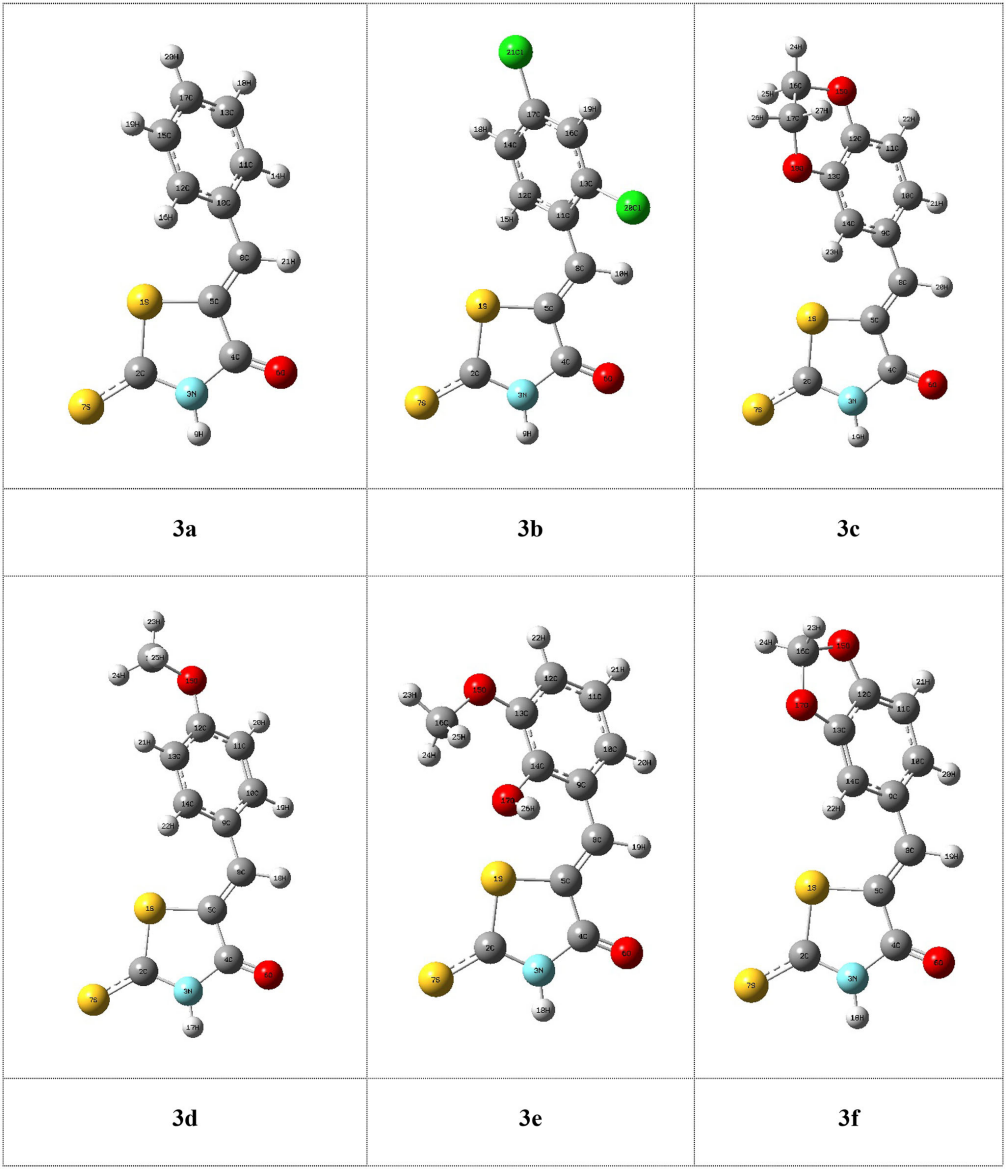

**Figure:**
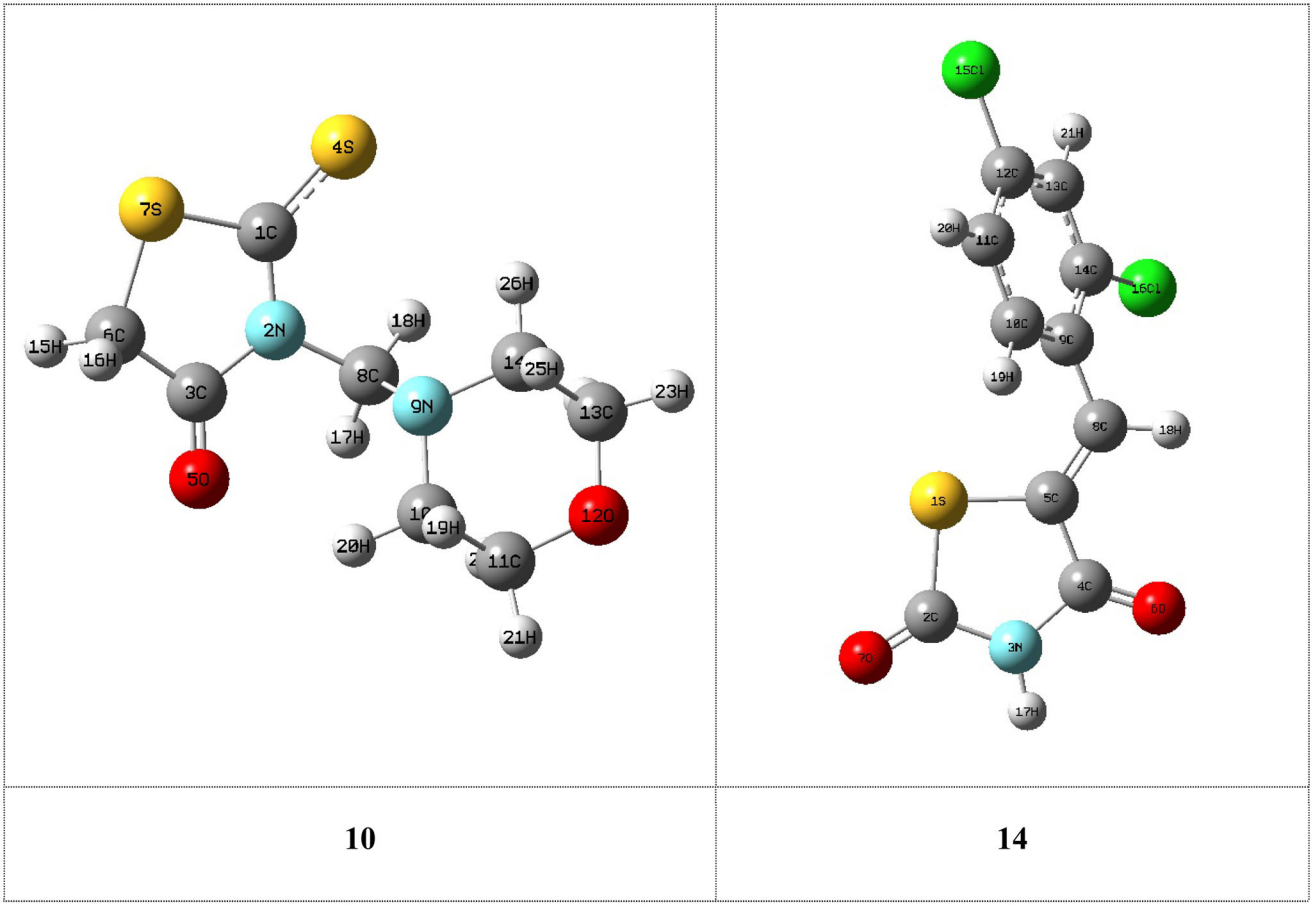

**Figure 2.**
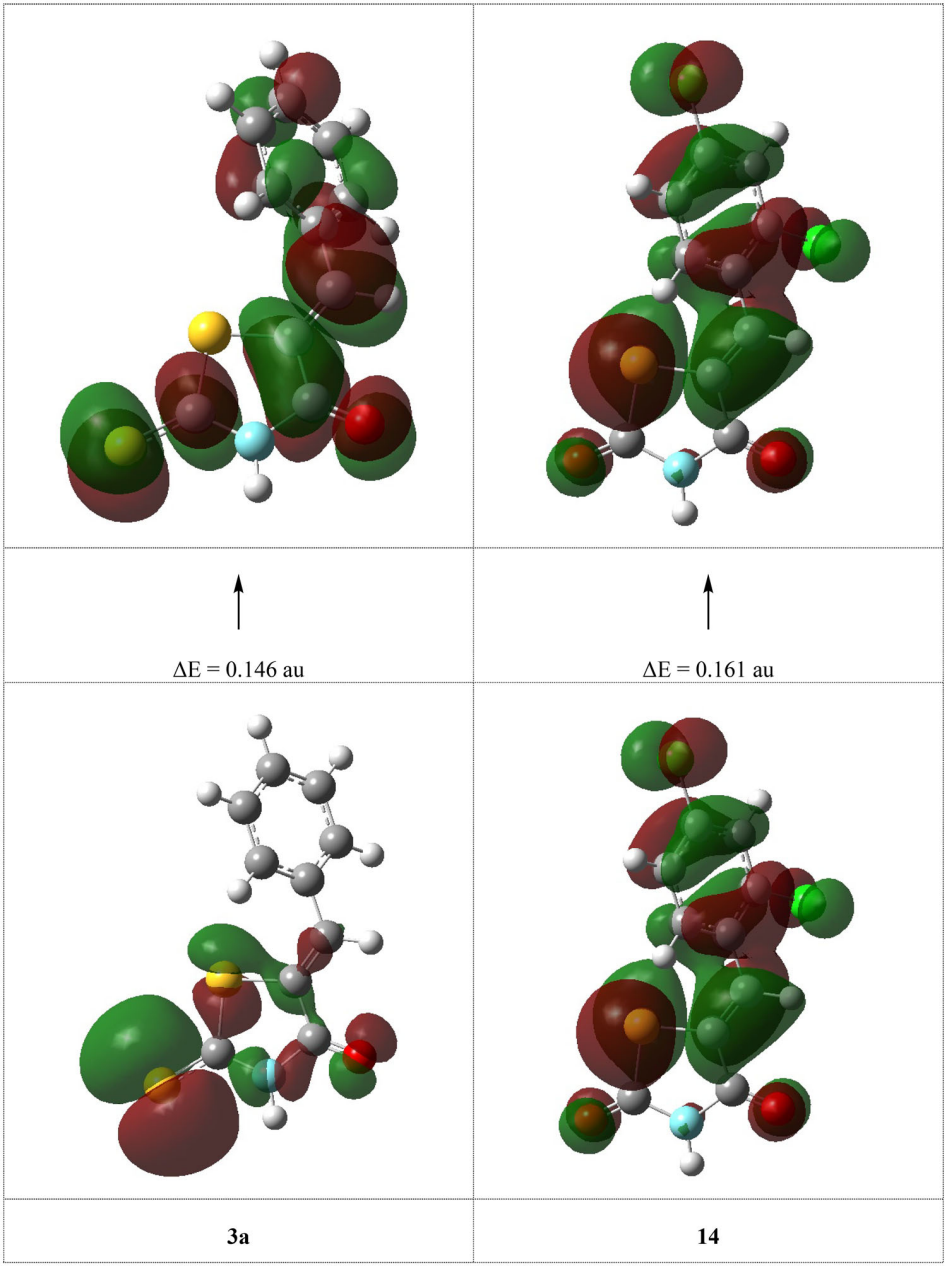
The calculated electronic transition between HOMO-LUMO for the highest and lowest biologically active **3a** and **14** inhibitors.

The interaction between the HOMO and LUMO levels of the reacting species determines chemical reactivity, according to the frontier molecular orbital theory, or FMO. The EHOMO and ELUMO symbols on a molecule represent its capacity to give electrons to an applicable acceptor with vacant molecular orbitals and its capacity to accept electrons, respectively. The ability of the molecule to receive electrons increases with the value of ELUMO. Comparison the reactivity between compound **3a** and **3d**, the insertion of methoxy group substituent increase the energy of the HOMO of **3d** inhibitor by 0.017 au, which means that this compound could react as nucleophile (electron donor), Table 1. Also, many theoretical models for describing the structure and conformation barriers in molecular systems make use of the HOMO-LUMO energy gap, E, which is an important stability index. For a given molecule, the lower its value of E, the greater the likelihood that it possesses inhibitory efficiency. The calculations showed that inhibitor **3d** has a smaller ΔE (0.132 au) than that of **3a** (0.146 au), Figure 2, which means increasing the biological activity for **3d** compound which agree well with experimental observations, Table 1. The dipole moment, D, the first derivative of the energy with respect to an applied electric field, was used to discuss and rationalise the structure. There is a good correlation between D and inhibition efficiency. This was shown from increasing the dipole moment. The molecule with higher efficiency, compound **3d**, has higher dipole moment (5.229 D) than that of compound 3a (3.738 D) (Table 1).

**Table 1.** The calculated quantum chemical parameters obtained from DFT/B3LYP/6-31 + G (d) of the investigated compounds.

Compound	HOMO	LUMO	ΔE	Dipole	IP	EA	Η	σ	µ	χ	E_t_
	(au)	(au)	(au)	(D)	(au)	(au)	(au)	(au^-1^)	(au)	(au)	(au)
**3a**	−0.236	−0.090	0.146	3.738	0.236	0.090	0.076	13.158	−0.163	0.163	−1304.868
**3b**	−0.243	−0.099	0.144	2.191	0.243	0.099	0.072	13.889	−0.172	0.172	−1418.769
**3c**	−0.218	−0.084	0.134	5.337	0.218	0.084	0.067	14.925	−0.151	0.151	−1531.461
**3d**	−0.219	−0.087	0.132	5.229	0.219	0.088	0.065	15.385	−0.153	0.153	−1418.769
**3e**	−0.218	−0.089	0.129	4.616	0.219	0.089	0.065	15.384	−0.154	0.154	−1493.536
**3f**	−0.218	−0.087	0.131	4.220	0.219	0.087	0.066	15.152	−0.153	0.153	−1492.352
**6**	−0.235	−0.069	0.166	11.057	0.235	0.069	0.083	12.048	−0.152	0.152	−2251.826
**7**	−0.224	−0.043	0.181	5.089	0.224	0.043	0.091	11.050	−0.133	0.133	−2251.792
**10**	−0.207	−0.065	0.142	2.629	0.207	0.065	0.071	14.085	−0.136	0.153	1361.318
**13a**	−0.229	−0.077	0.152	3.834	0.229	0.077	0.076	13.158	−0.153	0.153	−2519.454
**13b**	−0.246	−0.096	0.150	1.167	0.246	0.096	0.075	13.333	−0.171	0.171	−334.258
**13c**	−0.213	−0.071	0.142	9.560	0.213	0.071	0.071	14.085	−0.142	0.142	−2746.060
**14**	−0.248	−0.087	0.161	1.350	0.248	0.087	0.080	12.500	−0.167	0.167	−1898.228

Absolute hardness, η, and softness, σ are important properties that measure both the stability and reactivity of a molecule. The energy gap between a hard molecule and a soft molecule is considerable for the former and small for the latter. Because they are more likely to donate electrons to an acceptor, soft molecules are more reactive than their rigid counterparts. A biological system’s inhibitor serves as a Lewis base, whereas the enzyme plays the role of a Lewis acid. The increasing the softness and chemical potential of **3d** inhibitor (15.383 au^−1^, and −0.153 au) respectively could be responsible for increasing its biological activity more than that **3a** (13.158 au^−1^ and −0.163 au) respectively, Table 1, which agrees well with the experimental observations.

It could be observed from the calculations that the insertion of hydroxyl and methoxy groups on the oxothizlidine-4-one moiety, increases highly the reactivity of **3e** molecule with respect to **3a** inhibitor. This was shown by increasing the energy of HOMO, dipole, softness and decreasing the ΔE, and electronegativity (−0.219 au, 0.130 au, 4.616 D, 15.384 au^−1^ and 0.154 au) respectively, Table 1.

To compare the effect of dioxolane, **3f**, and dioxane, **3c**, substituents on the biological activity of inhibitor 3a, the calculations showed that inhibitors **3f** and **3c** have higher activity than that of unsubstituted **3a** inhibitor. Moreover, the calculations showed that the dioxolane substituent has higher reactivity than that of dioxane substituent. This was shown from the decreasing the energy of the LUMO (−0.087 au) which means that **3f** inhibitor has more electron accepting ability from enzyme than that of **3c** compound (-0.084 au), Table 1. Also, **3f** has a lower energy gap, ΔE (0.131 au), than that of **3c** (0.134) which could be responsible for increasing the reactivity of **3f** more that of **3c**. Meanwhile, increasing the softness and chemical potential of compound **3f** (15.152 au^−1^ and −0.153 au, respectively) could increase its reactivity with respect to **3c** with dioxane substituent. This is in a good agreement with the experimental data.

Meanwhile, one can see that the presence of dichloro-substituent on thiazlolidine-4-one moiety is slightly increased the reactivity of compound **3b** with respect to compound **3a**. This could be attributed to increasing the electron affinity, softness and electronegativity of **3b** inhibitor (0.099 au, 13.889 au^−1^ and 0.172 au, respectively) more than that of **3a** inhibitor (0.090 au, 13.158 au^−1^ and 0.163 au, respectively). Also, the lowering of ΔE value, which is a function of reactivity, for **3b** (0.144 au) more than that of **3a** (0.146 au) could be responsible for increasing the its reactivity towards the enzyme, Table 1, which is in agreement with experimental observation.

Experimental observations showed that the replacement of sulphur atom in thiazolidine-2,4-dione moiety by oxygen atom is highly decreased the biological activity of inhibitor **14** with respect to inhibitor **3a**. This was confirmed from the calculations which showed the effect of substituent on the calculated quantum chemical parameters. The calculations showed the highly increasing the energy gap between HOMO-LUMO of inhibitor **14** (0.161 au) which means increasing its stability (less reactive) more than that of 3a (0.146 au), Figure 2. Also, the decreasing the energy of HOMO, dipole moment, electron affinity and softness (−0.248 au, 1.350 D, 0.087 au, and 12.500 au^−1^, respectively) and increasing the electronegativity (0.167) could be responsible for the decreasing the reactivity of inhibitor 14 and accordingly decreasing its biological activity which is in a good agreement with the experimental observation.

Surprisingly, the morpholinomethyl-2-thioxothiazolidin-4-one, inhibitor **10**, was found experimentally to be biological inactive which was confirmed from the calculated quantum parameters. The calculations showed that compound 10 has high energy gap, ΔE (0.142 au), low electron affinity (0.065 au) which probably responsible for biological inactive which agrees well with experimental data.

From the above, we could conclude the importance of computational chemistry to explain the reactivity of the investigated molecules and accordingly the biological activity.

### Compounds 6 and 7

**Figure 3.**
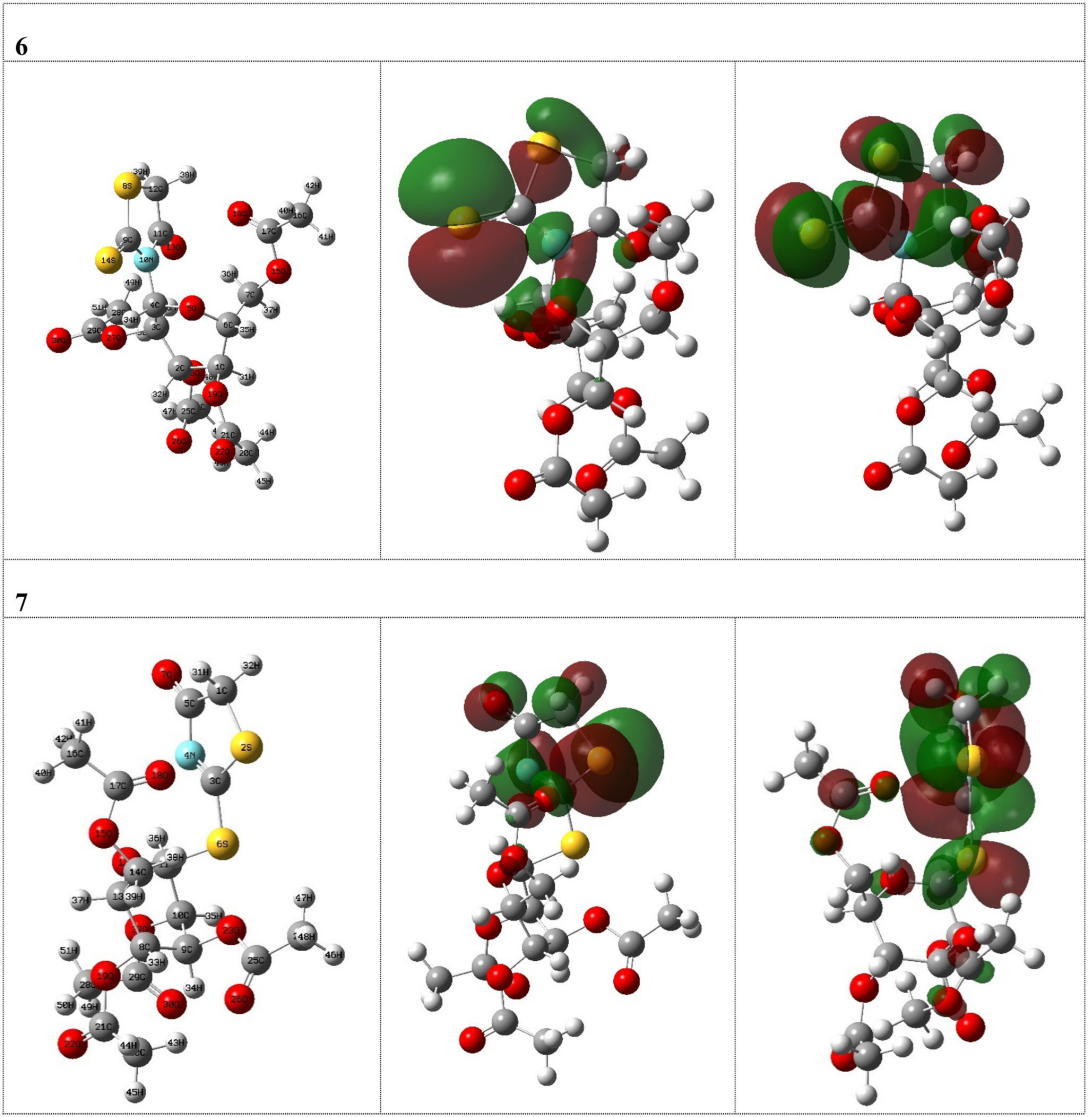
The optimised molecular structures, charge density distributions (HOMO and LUMO) for the investigated compounds **6** and **7**.

Quantum chemical calculations were performed to investigate the effect of structural parameters on the biological activity of the investigated compounds **6** and **7**. It was shown from the calculations that the geometrical structures of the investigated organic compounds are not planner. The optimised molecular structures with minimum energies obtained from the calculations of the investigated compounds are shown in Figure 3.

We started our calculations to make a comparison between the stability of the investigated compounds **6** and **7** and the calculations showed that compound **6** is more stable than compound **7** by about 0.035 au. which is in a good agreement with the experimental observations.

Also, the experimental data showed that the presence of sugar moiety at the nitrogen atom of thioxothiazoline-4-one moiety, compound **6**, showed a higher biological activity than that at sulphur atom of the same moiety, compound **7**.

The calculations showed that the efficient inhibitor **6** has a lower energy for LUMO (−0.069 au) which increase its ability to accept charges from the enzyme and accordingly increase its biological activity, which is in a good agreement with the experimental observations. It was shown from the calculations that the inhibitor 6 has the smaller HOMO–LUMO gap (0.166 au) compared with the that of molecule **7** (0.181 au), Table 1. Accordingly, it could be expected that the molecule **6** has more inclination to interact with reactivity higher than that of molecule **7** which agrees well with the experimental data.

The dipole moment, D, was used to discuss and rationalise the structure. There is a good correlation between D and inhibition efficiency. The molecule with higher efficiency, compound **6**, has higher dipole moment (11.057 D) than that of compound **7** (5.089 D) (Table 1).

Absolute hardness, η, and softness, σ are important indices which measure both the stability and reactivity of a molecule. Accordingly, it was concluded from the calculations that inhibitor **6** with higher σ value (12.048 au^−1^), has higher ability inhibition efficiency compared to inhibitor **7** (11.050 au^−1^), Table 1, which is in a good agreement with the experimental data. Also, the calculations showed that inhibitor 6 has higher χ (0.152 au), Table 1, which leads to increase its donation ability to the enzyme and accordingly enhance its inhibition efficiency. It is concluded from the above discussion that the quantum chemical parameters confirm that the inhibitor with sugar substituents on nitrogen atom of thioxothiazoline-4-one moiety has higher inhibition efficiency than that on sulphur atom which agrees well with the experimental observations.

Additionally, the HOMO and LUMO levels—two widely used quantum chemical parameters—have an impact on how a molecule interacts with other molecules. Figure 3 displays the charge density distribution for the investigated compounds at the HOMO and LUMO level. The studied compounds revealed that the HOMO levels, which could react with the biological target as a nucleophile (hydrogen bond acceptor), are primarily localised on the thioxothiazolidine-4-one moiety with the lone pairs of sulphur, oxygen, and nitrogen atoms, indicating that these moieties are the preferred sites for the attacking electrophile at the enzyme. The LUMO, which could be reacted as an electrophile (hydrogen bond donor) with the biological target is also completely localised on localised on the thioxothiazoline-4-one moiety as antibonding n-π*character of C-S, C-N and C-O groups which means that these moieties could be reacted as electrophile (electron acceptor). Surprisingly, the calculations showed that there is no contribution at all for sugar moiety which means that the insertion of sugar moiety will not effect on the biological activity.

### Compounds 13a–c

**Figure 4.**
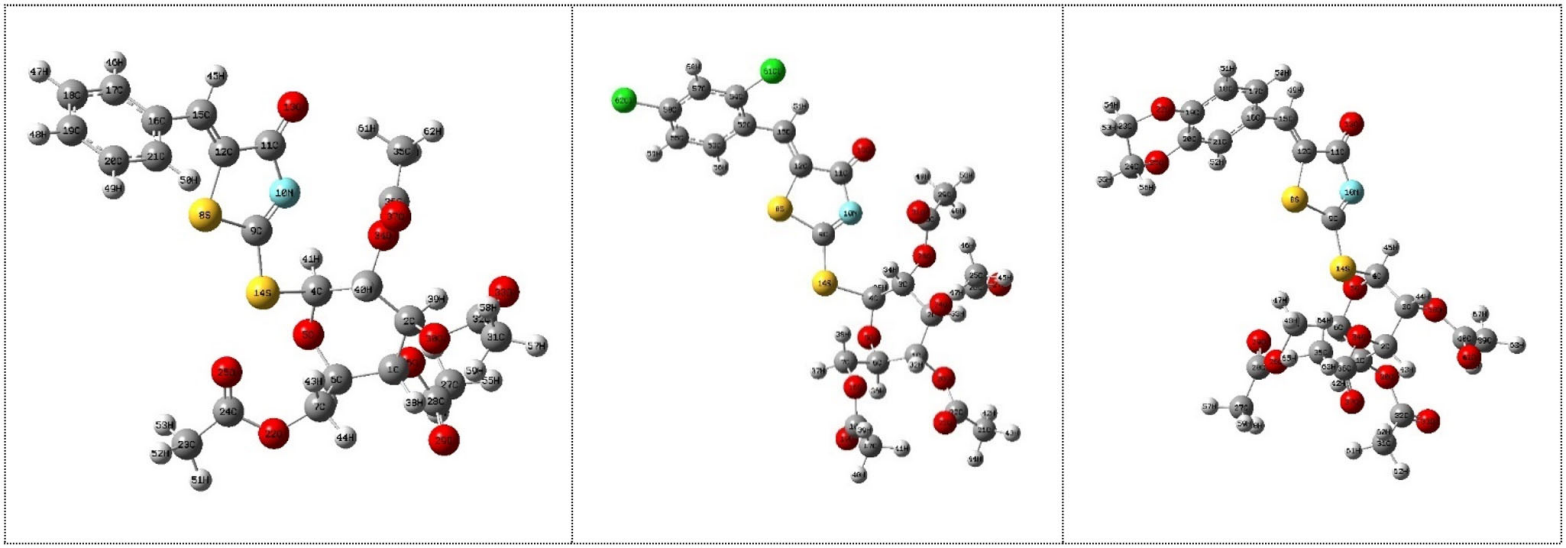
The calculated molecular structures of the investigated compounds **13a**, **13b**, and **13c**.

The DFT molecular modelling calculations were performed to study the effect of inserion the sugar moiety on the sulphur atom of the thiooxothiazolin-4-one on the inhibition efficiency of the investigated compounds for enzyme. The molecular structures of the investigated compounds **13a**, **13b** and **13c** obtained from the calculations are shown in Figure 4. By comparing between the inhibition of **3a**, **3b** and **3c** compounds with that of **13a**, **13b** and **13c** compounds, the calculations showed that the presence of sugar moiety decreases the biological activity which is in a good agreement with the experimental observations. Surprisingly, the calculations showed that inhibitors 3a, 3 b, and 3c have the lower energies of LUMO (−0.090, −0.099, and −0.094 au, respectively) than that of **13a**, **13b** and **13c** (−0.077, −0.096, and −0.071 au, respectively), which means that these compounds could react as electrophiles (electron acceptor) with enzyme, Table 1. Also, the decreasing the energy gap, ΔE, between HOMO-LUMO for compounds **3a**, **3b**, and **3c** (0.146, 0.144, and 0.134 au, respectively), means that those inhibitors are probably more favourable for the reactivity towards the enzyme. The increasing of the chemical potential, electronegativity and mean increasing the reactivity of the molecule and accordingly increase the biological activity, which agrees well with the experimental observations (Table 1).

Absolute hardness, η, and softness, σ are important indices that measure both the stability and reactivity of a molecule. Accordingly, it was concluded that inhibitors (**3a**, **3b**, and **3c**) with higher σ value (13.158, 13.884, and 14.925 au^−1^, respectively) have higher ability inhibition efficiency compared to inhibitors **13a**, **13b**, and **13c** (13.158, 13.333, and 14.885 au^−1^, respectively), Table 1, which is in a good agreement with the experimental data. Also, the calculations showed that inhibitors **3a**, **3b**, and **3c** have higher χ (0.153, 0.172 and 0.151 au, respectively), which leads to increase its donation ability to the enzyme and accordingly enhance its inhibition efficiency. It was concluded from the above discussion that the quantum chemical parameters confirm that the inhibitor with sugar substituents on nitrogen atom of thioxothiazoline-4-one moiety has higher inhibition efficiency than that on sulphur atom which agrees well with the experimental observations.

## Biological activities

The synthesised compounds were screened for their cytotoxicity against breast, liver and lung cancer cells using the MTT assay. As seen in Table 2, most exhibited good cytotoxicity against MCF-7, hepG2 and A549 cells with promising IC_50_ values. Interestingly, compounds **6, 7** and **13a** showed potent cytotoxicity with IC_50_ values of (11.7, 0.21, 1.7 µM), (12.4, 0.76, 0.31) and (3.1, 17.2, 6.1), respectively compared to Dox (7.67, 8.28, 6.62 µM). While some compounds were found to be not active with IC_50_ values higher than 50. Hence, these compounds **6, 7** and **13a** were investigated for molecular target and apoptosis activity.

**Table 2.** Cytotoxic IC_50_ values of the tested compounds against MCF-7, HepG2 and A549 cell lines using the MTT assay.

Code (AIK)	IC_50_ µM		
	MCF-7	HepG2	A549
**3a**	2.1	8.2	17.1
**3b**	17.9	7.8	9.7
**3c**	7.1	11.7	13.7
**3d**	15.1	18.1	7.8
**3e**	3.1	16.3	7.7
**3f**	12.8	8.9	21.1
**6**	11.7	0.21	1.7
**7**	12.4	0.76	0.31
**10**	ND	ND	36.4
**13a**	3.1	17.2	6.1
**13b**	13.1	7.2	10.1
**13c**	ND	21.2	4.1
**14**	42.2	37.3	31.6
**Doxorubicin**	7.67	8.28	6.62

ND: Not Determined.

### Topo II and DNA intercalation assay

Compounds **6, 7** and **13a** with the highest cytotoxic activity against HepG2 cells were tested against the Topo II and DNA intercalation activities to highlight their mechanistic study. As seen in Table 3, the tested compounds exhibited promising dual Topo II and DNA intercalation activities, interestingly, compound **6** had IC_50_ values of 6.9 and 19.6 µM, respectively compared to Dox (IC_50_ = 9.65 and 31.27 µM). Additionally, compounds **13a** and **7** exhibited promising inhibition activity with IC_50_ values of 8.3 and 9.1 µM against Topo II and 22.6 and 29.6 µM against DNA intercalation. Hence, compound **6** was further investigated for apoptotic cell death in HepG2 cells.

**Table 3.** IC_50_ values of Topoisomerase II inhibition and DNA intercalation of the tested compounds.

Compound	IC_50_^a ^[µM]	
	Topoisomerase II	DNA intercalation DNA/methyl green
**6**	6.9	19.6
**7**	9.1	29.6
**13a**	8.3	22.6
**Doxorubicin**	9.65	31.27

^a^**“**Values are expressed as average of three independent replicates”. “IC_50_ values were calculated using sigmoidal non-linear regression curve fit of percentage inhibition against five concentrations of each compound”.

### Apoptotic investigation of compound 6 against HepG2

**Figure 5.**
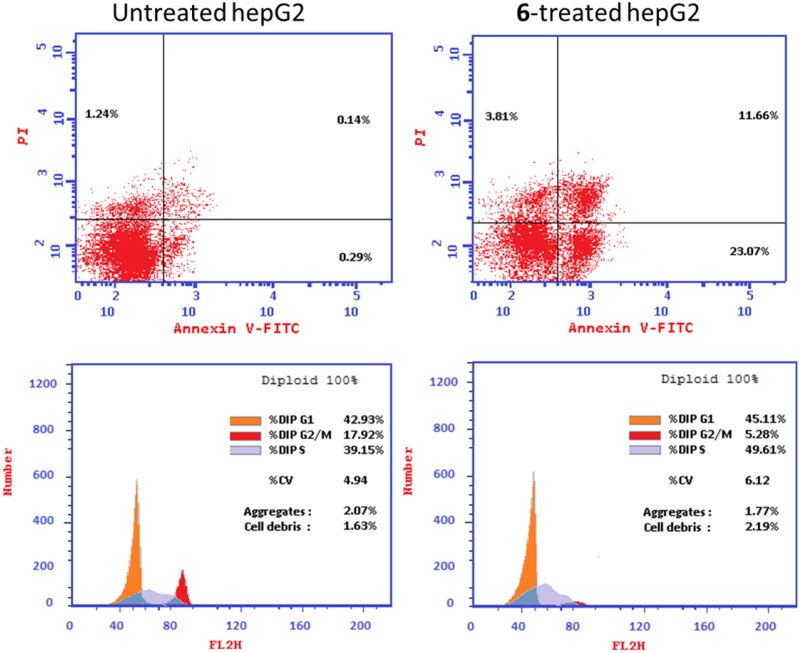
(A) Cryptographs of annexin-V/Propidium Iodide staining of untreated and **6-**treated HepG2 cells with the IC_50_ values, 48 h, “Q1-UL (necrosis, AV-/PI+), Q1-UR (late apoptotic cells, AV+/PI+), Q1-LL (normal cells, AV-/PI-), Q1-LR (early apoptotic cells, AV+/PI-)”, (B) Percentage of cell population at each cell cycle G0-G1, S, G2/M, and Pre-G1 using DNA content-flow cytometry aided cell cycle analysis.

Flow cytometric examination of Annexin V/PI staining was used to examine apoptotic cell death in untreated and treated HepG2 cells to determine the apoptotic activity of compound **6** (IC_50_ = 0.21 M, 48 h). Figure 5(A) showed compound **6** significantly activated apoptotic cell death in HepG2 cells by 80.7-fold, it induced total apoptosis by 34.73% (23.07% for early apoptosis, 11.66% for late apoptosis) compared to the untreated control group (0.43%).

Afterward, DNA flow cytometry was used to determine the cell population in each cell phase following treatment with a cytotoxic agent. As seen in Figure 5(B), compound **6** treatment significantly increased the cell population at the S-phase by 49.6% compared to control 39.15%, while cells in G1 weren’t significantly increased, and cells in G2/M were decreased. Consequently, compound **6** induced apoptsis in HepG2 cells arresting the cell proliferation at S-phase.

### RT-PCR of apoptosis-inducing agents against HepG2

For validating the apoptotic cell death in HepG2 cells upon treatment with compound **6**, gene expression level using RT-PCR was investigated for the apoptosis-mediaged genes of P53, Bax, caspase-3,8,9 and Bcl-2 in both untreated and treated HepG2 cells. As seen in Table 4, compound **6**, upregulated the apoptosis-related genes by 6.16, 5.7, 8.3, 3.29, 6.14-fold change, while it inhibted the Bcl-2 expression by 0.36-fold. Hence, these findings indicated the apoptosis-mediated cell death of compound **6** treatment through intrinsic and extrinsic pathways.

**Table 4.** Fold of change of apoptosis-related genes in untreated and treated HepG2 cells.

Sample	Fold Change = 2^^-ΔΔCT^					
	Proapoptotic gene					Anti-apoptotic gene
	P53	Bax	Casp-3	Casp-8	Casp-9	Bcl-2
**6**-treated HepG2	6.16 ± 0.69	5.7 ± 0.26	8.3 ± 0.37	3.29 ± 0.5	6.14 ± 0.19	0.36 ± 0.01
Untreated HepG2	1					

“Values are expressed as Mean ± SD of three independent replicates. Data were normalized using β-actin as house-keeping gene”.

### Molecular docking

**Figure 6.**
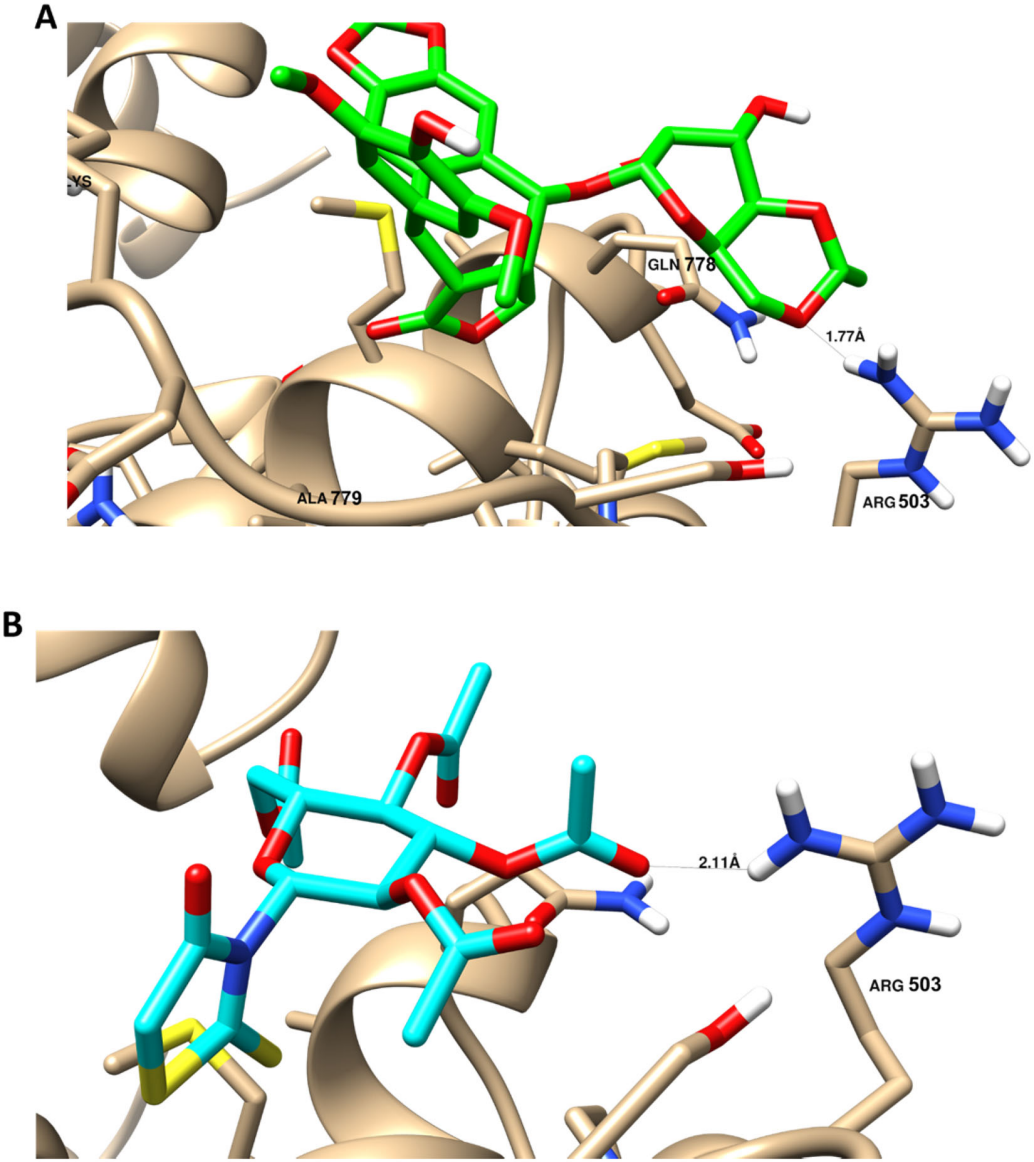
Binding disposition and ligand-receptor interactions of the co-crystallised ligand Etoposide (A); (Green-colored) and the docked compound **6** (B) (Cyan-colored) inside the topoisomeasre II (PDB = 3QX3) with the highlighted key amino acids.

Molecular docking study was conducted to highlight the mechanism of binding of the promising compound **6** against topoisomerase II enzymes, as they are important molecular drug targets and inhibitors of these enzymes are widely used as effective anticancer agents. Molecular docking using AutoDock vina software was validated by calculating the RMSD value to be 1.87 Å, and this was seen by overlying the co-crystallised ligand structuers in the self-docking. As seen in Figure 6, binding disposition and ligand receptor interacion of compound **6** inside the protein active site with active amino acids of Arg 503, Gln 778, and Ala 779. Compound **6** was properly docked with binding energy of −11.31 Kcal/mol, and it formed a good binding interactions with Arg 503 as key amino acid.

## Conclusion

We have carried out the successful synthesis of hitherto unreported 3-(2′,3′,4′,6′-tetra-*O*-acetyl-β-D-glucopyranosyl)-2-thioxo-4-thiazolidinone (**6**), 2–(2′,3′,4′,6′-tetra-*O*-acetyl-β-D-glucopyranosyl)-2-thioxo-4-thiazolidinone (**7**), 3-(β-D-glucopyranosyl)-2-thioxo-4-thiazolidinones (**8**) and their corresponding 5-(*Z*)-arylidene derivatives (**13a–c**). The conformational analyses of their most stable configurations were established by NMR spectroscopy. The antiviral and other antitumor activities of the new prepared compounds are being investigated and will be reported in due course. The nucleoside base **1** can be used as a starting material for the synthesis of other carbohydrate derivatives such as deoxy, amino and azido nucleosides. To examine the stable structure of the compounds, DFT calculations using the B3LYP/6-31 + G (d,p) level were used to infer the electronic and geometric structures. The calculated quantum chemical parameters and the experimental findings exhibited a strong correlation. Interestingly, compounds **6** exhibited promising cytotoxicity against MCF-7, HepG2 and A549 cells with IC_50_ values of 11.7, 0.21, and 1.7 µM, compared to Dox 7.67, 8.28, and 6.62 µM, respectively. For the molecular target, compound **6** exhibited topoisomerase II inhibition and DNA intercalation with IC_50_ values of 6.9 and 19.6 µM, respectively compared to Dox. Additionally, compound **6** treatmnet significantly activated apoptotic cell death in HepG2 cells by 80.7-fold, arresting the cell cycle at the S-phase by 49.6% Hence, glucosylated Rhodanines may serve as a promising drug candidates against cancer through apoptosis with promising topoisomerase II and DNA interchelation.

## Experimental section

### Chemistry and analysis

Melting points have been identified on the Büchi apparatus and have not been corrected. TLC was performed on a silica gel of aluminium 60 F_254_ (Merck) sheet, and detected by short UV light. Infra-red spectra of potassium bromide pellets were obtained using a Pye Unicam 1000 spectrometer. ^1^H NMR and ^13^C NMR spectra were measured on a 300 MHz Bruker Advance DPX spectrophotometer in DMSO-d_6_ or CDCl_3_ using TMS as an internal standard. Chemical shifts are given in ppm and *J* in Hz. Analytical data were acquired with a C, H, N Elemental Carlo Erba 1106 analyser. Mass spectra were recorded by EI on a Varian MAT 112 spectrometer and FAB on a Kratos MS spectrometer.

#### General procedures for the synthesizing 3a–3f

The nucleoside bases **3a–f**, were prepared form the direct condensation of **1** with the appropriate aromatic aldehydes (**5a–f**) according to our previous reported procedure with using morpholine instead of piperidine in the previous method^70^ as the following: To a mixture of 2-thioxo-4-thiazolidinone (**1**) (1.33 g, 10 mmol), anhydrous morpholine (0.87 g, 10 mmol) and anhydrous EtOH (30 ml), appropriate aromatic aldehydes (**2a–f**) were added (11 mmol). The mixture was stirred until the starting material was consumed (12 h; TLC). The reaction mixture was diluted with water and neutralised with dilute hydrochloric acid. The yellow solid that was separated by filtration was collected and recrystallized from ethanol to give products **3a–f** in quantitative yields.

#### 5-((Z)-benzylidene)-2-thioxo-4-thiazolidinone (3a)

Yield 2.10 g (95%); mp 208–210 °C (lit.^69^ yield 90%, mp 204–206 °C); IR (KBr): *v* 3190 (NH), 1726 (C=O) cm^−1^; ^1^H NMR (300 MHz, DMSO-d_6_): δ 7.49–7.59 (m, 5H, Ar-H), 7.62 (s, 1H, =CH), 13.85 (s, 1H, NH); ^13^C NMR (300 MHz, DMSO-d_6_): δ 125.90 (=CH), 129.84, 130.90, 131.14, 132.05, 133.36 (C-5, C-Ar), 169.76 (C-4), 196.07 (C-2).

#### 5-((Z)-2,4-dichlorobenzylidene)-2-thioxo-4-thiazolidinone (3b)

Yield 2.84 g (98%); mp 230–232 °C (lit.^66^ yield 90%, mp 228–230 °C); IR (KBr): *v* 3190 (NH), 1723 (C=O) cm^−1^; ^1^H NMR (300 MHz, DMSO-d_6_): δ 7.43–7.76 (m, 4H, =CH, Ar-H), 13.86 (s, 1H, NH); ^13^C NMR (300 MHz, DMSO-d_6_): δ 124.56 (=CH), 125.37, 128.33, 129.66, 129.88, 129.89, 130.23, 135.66 (C-5, C-Ar), 169.06 (C-4), 195.07 (C-2); MS, *m/z* 289 (M^+^).

#### 5-((Z)-3,4-ethylenedioxybenzylidene)-2-thioxo-4-thiazolidinone (3c)

Yield 2.60 g (93%); mp 208–210 °C (lit.^70^ yield 94%, mp 207– 208 °C); IR (KBr): *v* 3196 (NH), 1736 (C=O) cm^−1^; ^1^H NMR (300 MHz, DMSO-d_6_): δ 4.26 (m, 4H, 2 x OCH_2_), 6.94 (m, 3H, Ar-H), 7.38 (s, 1H, =CH) 13.65 (s, 1H, NH); ^13^C NMR (300 MHz, DMSO-d_6_) d 64.14, 64.71 (2 x OCH_2_), 118.19, 119.35, 122.99, 124.57, 125.69, 126.40, 131.79, 143.89, 146.15 (C-Ar, =CH, C-5), 169.52 (C-4), 195.46 (C-2); MS, *m/z* 279 (M^+^).

#### 5-((Z)-4-methoxybenzylidene)-2-thioxo-4-thiazolidinone (3d)

Yield 2.44 g (97%); mp 257–259 °C (lit.^71^ yield 90%, mp 254– 256 °C); IR (KBr): *v* 3194 (NH), 1728 (C=O) cm^−1^; ^1^H NMR (300 MHz, DMSO-d_6_): δ 7.13 (d, *J* = 8.76 Hz, 2H, 2′-H, 6′-H), 7.59 (d, *J* = 8.86 Hz, 2H, 3′-H, 5′-H), 7.63 (s, 1H, =CH), 13.76 (s, 1H, NH); ^13^C NMR (300 MHz, DMSO-d_6_): δ 125.90 (=CH), 129.84, 130.90, 131.14, 132.05, 133.36 (C-5, C-Ar), 169.76 (C-4), 196.07 (C-2).

#### 5-((Z)-(2-hydroxy-3-methoxybenzylidene)-2-thioxo-4-thiazolidinone (3e)

Yield 2.40 g (90%), mp 199–201 °C (lit.^70^ yield 86%, mp 197–198 °C); IR (KBr): *v* 3189 (NH), 1726 (C=O) cm^−1^; ^1^H NMR (300 MHz, DMSO-d_6_): δ 3.86 (s, 3H, OMe), 6.86–7.15 (m, 3H, Ar-H), 7.94 (s, 1H, =CH), 9.89 (s, 1H, OH), 13.77 (s, 1H, NH); ^13^C NMR (300 MHz, DMSO-d_6_): δ 56.23 (OMe), 114.43, 119.99, 120.50, 124.70, 127.19, 147.19, 148.26 (=CH, C-5, C-Ar), 170.10 (C-4), 196.43 (C-2); MS, *m/z* 267 (M^+^).

#### 5-((Z)-3,4-methylenedioxybenzylidene)-2-thioxo-4-thiazolidinone (3f)

Yield 2.45 g (92%); mp 211–212 °C (lit.^61^ yield 71%, mp 278– 279 °C); IR (KBr): *v* 3194 (NH), 1732 (C=O) cm^−1^; ^1^H NMR (300 MHz, DMSO-d_6_): δ 6.13 (s, 2H, OCH_2_O), 7.12 (m, 3H, Ar-H), 7.56 (s, 1H, =CH) 13.67 (s, 1H, NH).

#### General procedures for the synthesizing 6 and 7

2-Thioxo-4-thiazolidinone (**1**) (665 mg, 5 mmol) was suspended in anhydrous acetonitrile (25 ml) and BSA (1.25 ml, 5 mmol) was added, and the reaction mixture was heated at 70–80 °C for 30 min. 1,2,3,4,6-Penta-O-acetyl-α-D-glucopyranose (**5**) (1.87 g, 5 mmol) dissolved in anhydrous acetonitrile (25 ml) was added to the reaction mixture *via* a cannula. Finally, TMSOTf (1.00 ml, 5 mmol) was added, and the reaction mixture was heated at 70–80 °C for 60 min. Saturated NaHCO_3_ was added to quench the reaction and the resulting mixture was extracted with CH_2_Cl_2_. The collected organic fractions were washed with saturated NaCl solution, dried with MgSO4, filtered, and evaporated to dryness. The resulting solid was purified using flash chromatography (eluent 10–50%, ether/petroether ether, 40–60 °C) to give 6 and 7, respectively.

#### 3-(2',3',4',6'-Tetra-O-acetyl-β-D-glucopyranosyl)-2-thioxo-4-thiazolidinone (6)

This compound was separated as white foams; yield 0.14 g (6%); IR (KBr): *v* 1755 (CO), (CO), 1746 1238 (CS) cm^−1^; ^1^H NMR (300 MHz, CDCl_3_): δ 1.98, 2.04, 2.06, 2.10 (4 s, 12H, 4Ac), 3.88 (m, 3H, 5-H, H-5′), 4.23 (m, 2H, 6′-H, 6”-H), 5.25 (dd, *J* = 9.8, 9.8 Hz, 1H, 4′-H), 5.35 (dd, *J* = 9.4, 9.4 Hz, 1H, 2′-H), 5.92 (dd, *J* = 9.3, 9.8 Hz, 1H, 3′-H), 5.82 (d, *J* = 9.3 Hz, 1H, 1′-H); ^13^C NMR (300 MHz, CDCl_3_): δ 20.30, 20.40, 20.60, 20.74 (4Ac), 33.62 (C-5), 61.40 (C-6′), 67.50 (C-2′), 67.87 (C-3′), 72.82 (C-4′), 74.73 (C-5′), 82.43 (C-1′), 169.40, 169.90, 170.08, 170.67 (4Ac), 172.10 (C-4), 201.54 (C-2); EI ms: *m/z* = 463 (M^+^). Anal. Calcd. for C_17_H_21_NO_10_S_2_ (463.48): C, 44.05; H, 4.57; N, 3.02. Found: C, 44.24; H, 4.69; N, 2.83.

#### 2–(2',3',4',6'-Tetra-O-acetyl-β-D-glucopyranosylmercapto)-4-thiazolidinone (7)

This compound was separated as white foams; yield 0.70 g (30%); IR (KBr): *v* 1752 (CO) cm^−1^; ^1^H NMR (300 MHz, CDCl_3_): δ 2.02, 2.05, 2.07, 2.08 (4 s, 12H, 4Ac), 3.98 (m, 1H, 5′-H), 4.07 (s, 2H, H-5), 4.15 (dd, *J* = 2.1, 12.6 Hz, 6′-H), 4.31 (dd, *J* = 4.6, 12.6 Hz, 1H, 6”-H), 5.11 (dd, *J* = 9.6, 9.6 Hz, 1H, 4′-H), 5.35 (dd, *J* = 9.3, 10.2 Hz, 1H, 2′-H), 5.33 (dd, *J* = 9.3, 9.8 Hz, 1H, 3′-H), 5.82 (d, *J* = 10.4 Hz, 1H, 1′-H); ^13^C NMR (300 MHz, CDCl_3_): δ 20.06, 20.16, 20.31, 20.40 (4Ac), 39.14 (C-5), 61.13 (C-6′), 67.23 (C-2′), 69.74 (C-3′), 73.04 (C-4′), 75.94 (C-5′), 82.24 (C-1′), 169.01, 169.03, 169.45, 170.12 (4Ac), 186.53 (C-4), 198.83 (C-2); EI ms: *m/z* = 463 (M^+^). Anal. Calcd. for C_17_H_21_NO_10_S_2_ (463.48): C, 44.05; H, 4.57; N, 3.02. Found: C, 44.16; H, 4.78; N, 2.76.

#### General procedures for the synthesizing 3-(-β-D-glucopyranosyl)-2-thioxo-4-thiazolidinone (8)

The protected nucleoside **6** (463 mg, 1 mmol) was suspended in MeOH (15 ml) and concentrated HCl (0.5 ml) was added. The reaction mixture was stirred at 50 °C for 2 h, and the mixture was cooled to room temperature. A resin used for ion exchange (Amberlite IR-120, HO—form) that had been rinsed with MeOH was then added to the resultant solution. The reaction mixture was stirred for 5 min before being filtered and evaporated under vacuum, and the residue was purified by flash chromatography (eluent 0–5%, CHCl_3_/MeOH) to get 254 mg (86%) of **8**. This compound was separated as pale yellow foams; IR (KBr): *v* 3383 (OH), 1733 (CO), 1228 (CS) cm^−1^; ^1^H NMR (300 MHz, CDCl_3_): δ 3.13–3.66 (m, 6H, 5′-H, 6′-H, 4′-H, 3′-H, 2′-H), 4.60 (d, *J* = 4.6 Hz, 1H, 6′-OH), 4.78 (t, *J* = 5.2 Hz, 1H, 4′-OH), 5.20 (d, *J* = 5.5 Hz, 1H, 3′-OH), 5.32 (d, *J* = 6.0 Hz, 1H, 2′-OH), 5.67 (d, *J* = 10.1 Hz, 1H, 1′-H); ^13^C NMR (DMSO-d_6_): δ 33.40 (C-5), 61.74 (C-6′), 68.32 (C-2′), 70.54 (C-3′), 77.99 (C-4′), 81.08 (C-5′), 86.07 (C-1′), 175.14 (C-4), 206.55 (C-2); EI ms: *m/z* = 295 (M^+^). Anal. Calcd. for C_9_H_13_NO_6_S_2_ (295.33): C, 36.60; H, 4.44; N, 4.74. Found: C, 36.68; H, 4.57; N, 4.62.

#### General procedures for the synthesizing 2,4-thazolidinedione (9)

The protected nucleoside **7** (463 mg, 1 mmol) was suspended in MeOH (15 ml) and concentrated HCl (0.5 ml) was added. The reaction mixture was stirred at 50 °C for 2 h, and the mixture was cooled to room temperature. After washing with MeOH, ion exchange resin (Amberlite IR-120, HO—form) was added to the resultant solution. After 5 min of stirring, the mixture was filtered, evaporated *in vacuo*, and the residue was purified by flash chromatography (eluent 0–5%, CHCl3/MeOH) to get 167 mg (94%) of **9**. This compound has mp 125–127 °C (lit.^74^ yield 57%, mp 124–126 °C); IR (KBr): *v* 3198, CO 1756, CO 1710 cm^−1^; ^1^H NMR (300 MHz, DMSO-d_6_): δ 4.13 (s, 2H, CH_2_), 12.22 (s, 1H, NH); ^13^C NMR (300 MHz, DMSO-d_6_): δ 37.50 (C-5), 167.35 (C-2), 170.92 (C-4); EI ms: *m/z* = 177 (M^+^). Anal. Calcd. for C_3_H_3_NO_2_S (177.12): 30.77; H, 2.58; N, 11.96. Found: 30.89; H, 2.72; N, 11.63.

#### General procedures for the synthesizing 3-morpholinomethyl-2-thioxo-4-thiazolidinone (10)

A mixture of 2-thioxo-4-thiazolidinone (**1**) (665 mg, 5 mmol) and morpholine (87 mg, 1 mmol) in anhydrous ethanol (5 ml) and aqueous formaldehyde (0.1 ml) was stirred for 12 h at room temperature until the starting material was consumed (TLC). The separated solid was collected by filtration and recrystallized from ethanol to give 190 mg (82%) of **10** as a yellow solid. This compound has mp 104–106 °C; IR (KBr): *v* 1732 (CO), 1228 (CS) cm^−1^; ^1^H NMR (300 MHZ, CDCl_3_): δ 2.73 (t, *J* = 4.7 Hz, 4H, 2′-H, 6′-H), 3.64 (t, *J* = 4.7 Hz, 4H, 3′-H, 5′-H), 4.01 (s, 2H, 5-H), 4.92 (s, 2H, NCH_2_N); ^13^C NMR (300 MHZ, CDCl_3_): δ 35.26 (C-5), 51.70 (C-2′, C-6′), 65.29 (NCH_2_N), 66.78 (C-3′, C-5′), 175.06 (C-4), 203.18 (C-2); EI ms: *m/z* = 232 (M^+^). Anal. Calcd. for C_8_H_12_N_2_O_2_S_2_ (232.32): C, 41.45; H, 5.28; N, 11.87. Found: C, 41.36; H, 5.21; N, 12.06.

#### General procedures for the synthesizing 2-methylmercapto-4-thiazolidinone (11)

2-Thioxo-4-thiazolidinone (1) (1.33 g, 10 mmol) was dissolved in aqueous NaOH (2%, 25 ml) at room temperature. To this solution was added methyl iodide (1.56 g, 11 mmol), and the reaction mixture was stirred overnight at room temperature. The reaction mixture was diluted with CH_2_Cl_2_, washed with cold saturated aqueous NaHCO_3_ and water, and dried over anhydrous Na_2_SO_4_. CH_2_Cl_2_ was evaporated to dryness and the remaining methanol crystallised to yield 1.26 g (86%), melting point 72–74 °C (lit.^83^ 168 °C–170 °C).

#### General procedures for the synthesizing 13a-13c

To a mixture of the protected nucleoside **7** (0.46 g, 1 mmol), anhydrous morpholine (0.09 g, 1 mmol) and anhydrous ethanol (10 ml) was added the appropriate aromatic aldehydes (**12a–c**) (1 mmol). The mixture was stirred until the starting material was consumed (12 h; TLC). The reaction mixture was neutralised with HCl/MeOH. After stirring for 5 min, the solution was filtered and evaporated *in vacuo* and the residue was purified by flash chromatography (eluent 10–50%, ether/petroleum ether, 40–60 °C) to give the products **13a–c** as yellow solids.

#### 5-((Z)-benzylidene)-2–(2',3',4',6'-tetra-O-acetyl-β-D-glucopyranosyl)-2-thioxo-4-thiazolidinone (13a)

This compound has mp 166–168 °C; yield 0.49 (90%) IR (KBr): *v* CO 1750 cm^−1^; ^1^H NMR (300 MHZ, CDCl_3_): δ 2.03, 2.06, 2.07, 2.11 (4 s, 12H, 4Ac), 3.98–4.34 (m, 3H, 5′-H, 6′-H, 6”-H), 5.14–5.40 (m, 3H, 4′-H, 2′-H, 3′-H), 5.98 (d, *J* = 10.4 Hz, 1H, 1′-H), 7.45–7.53 (m, 5H, Ar-H), 7.91 (s, 1H, =CH); ^13^C NMR (300 MHZ, CDCl_3_): δ 20.21, 20.33 (4Ac), 61.26 (C-6′), 67.36 (C-2′), 68.92 (C-3′), 73.21 (C-4′), 76.12 (C-5′), 81.76 (C-1′), 125.10 (=CH), 125.63 (C-5), 129.05, 130.28, 130.84, 132.93, 137.03 (C-Ar), 169.10, 169.14, 169.50, 170.20 (4Ac), 178.82 (C-4), 189.22 (C-2); EI ms: *m/z* = 551 (M^+^). Anal. Calcd. for C_24_H_25_NO_10_S_2_ (551.59): C, 52.26; H, 4.57; N, 2.54. Found: C, 52.36; H, 4.67; N, 2.42.

#### 5-((Z)-2,4-dichlorobenzylidene)-2–(2',3',4',6'-tetra-O-acetyl-β-D-glucopyranosyl)-2-thioxo-4-thiazolidinone (13 b)

This compound has mp 167–169 °C; IR (KBr): *v* CO 1752 cm^−1^; ^1^H NMR (300 MHZ, CDCl_3_): δ 2.02, 2.05, 2.07, 2.11 (4 s, 12H, 4Ac), 3.96–4.36 (m, 3H, 5′-H, 6′-H, 6”-H), 5.13–5.42 (m, 3H, 4′-H, 2′-H, 3′-H), 5.98 (d, *J* = 10.4 Hz, 1H, 1′-H), 7.45–7.68 (m, 3H, Ar-H), 7.93 (s, 1H, =CH); EI ms: *m/z* = 620 (M^+^). Anal. Calcd. for C_24_H_23_Cl_2_NO_10_S_2_ (620.48): C, 46.46; H, 3.74; N, 2.26. Found: C, 46.54; H, 3.86; N, 2.20.

#### 5-((Z)-2,4-ethylenedioxybenzylidene)-2–(2',3',4',6'-tetra-O-acetyl-β-D-glucopyranosyl)-2-thioxo-4-thiazolidinone (13c)

This compound was separated as yellow foams; IR (KBr): *v* CO 1750 cm^−1^; ^1^H NMR (300 MHZ, CDCl_3_): δ 2.02, 2.04, 2.07, 2.09 (4 s, 12H, 4Ac), 3.76 (m, 1H, 5′-H), 4.07–4.30 (m, 6H, 6′-H, 6”-H, 2CH_2_), 4.88 (dd, *J* = 9.7, 9.7 Hz, 1H, 4′-H), 5.06 (dd, *J* = 9.3, 9.3 Hz, 2′-H), 5.54 (dd, *J* = 9.2, 9.2 Hz, 3′-H), 5.97 (d, *J* = 10.3 Hz, 1H, 1′-H), 6.93–7.07 (m, 3H, Ar-H), 7.80 (s, 1H, =CH); ^13^C NMR (300 MHZ, CDCl_3_): δ 20.33, 20.47, 20.50, 20.52 (4Ac), 61.82 (C-6′), 63.93, 64.50 (2 OCH_2_), 67.44 (C-2′, C-3′), 72.33 (C-4′), 73.35 (C-5′), 81.82 (C-1′), 118.08, 119.12, 122.83, 125.00, 126.46, 137.50, 143.84, 146.53 (C-Ar, =CH, C-5), 169.56, 170.06, 170.10, 170.56 (4Ac), 179.40 (C-4), 188.86 (C-2); EI ms: *m/z* = 609 (M^+^). Anal. Calcd. for C_26_H_27_NO_12_S_2_ (609.62.60): C, 51.22; H, 4.46; N, 2.30. Found: C, 50.94; H, 4.62; N, 2.17.

### General procedures for the synthesizing 5-((Z)-2,4-dichlorobenzylidene)-2,4-thiazolidindione (14)

#### Method A

To a stirred suspension of protected nucleoside **13b** (620 mg, 1 mmol) in anhydrous MeOH (15 ml) a portion of NaOMe (0.06 g, 1.1 mmol) in anhydrous MeOH (15 ml) was added at 0 °C. Then the reaction mixture was stirred for 2 h at room temperature. An ion exchange resin (Amberlite IR-120, H^+^-form) was added to the resulting solution, which was pre-washed with MeOH. After stirring for 5 min, the solution was filtered and evaporated *in vacuo* and the residue was purified by flash chromatography (0–5% elution, CHCl_3_/MeOH) to provide 214 mg (78%) of **14** as a pale yellow solid.

#### Method B

Protected nucleoside **13b** (620 mg, 1 mmol) was suspended in MeOH (15 ml) and concentrated HCl (0.5 ml) was added. The reaction mixture was heated for 2 h at 50 °C. An ion exchange resin (Amberlite IR-120, OH^-^-form) was added to the resulting solution, which was pre-washed with MeOH. After stirring for 5 min, the solution was filtered and evaporated *in vacuo* and the residue was purified by flash chromatography (0–5% elution, CHCl_3_/MeOH) to provide 255 mg (93%) of **14** as a pale yellow solid.

This compound has mp 206–208 °C; IR (KBr): *v* NH 3198, CO 1758, CO 1709 cm^−1^; ^1^H NMR (300 MHZ, DMSO-d_6_): δ 7.50–7.80 (m, 4H, Ar-H, =CH), 12.82 (s, 1H, NH); ^13^C NMR (300 MHZ, DMSO-d_6_): δ 125.35, 125.42, 127.76, 128.22, 129.82, 129.89, 129.94, 135.29, 135.37 (=CH, C-5, C-Ar), 166.79 (C-4), 167.27 (C-2); EI ms: *m/z* = 274 (M^+^). Anal. Calcd. for C_10_H_5_Cl_2_NO_2_S (274.12): C, 43.81; H, 1.84; N, 5.11. Found: C, 44.02; H, 2.00; N, 4.94.

## Biology

### Cytotoxicity against cancer cells

Using RPMI-1640 medium L-Glutamine (Lonza Verviers SPRL, Belgium, cat#12-604F), we successfully cultured liver (HepG2), breast (MCF-7), and lung (A549) cells bought from the National Research Institute, Egypt. Each culture was supplemented with 10% foetal bovine serum (FBS, Sigma-Aldrich, MO, USA) and 1% penicillin-streptomycin (Lonza, Belgium). Using a 96-well plate, cells were seeded at a density of 5,000 cells per well in triplicate. The chemicals were added to the cells on day 2 at doses of (0.01, 0.1, 1, 10, and 100 M). To determine cell viability, MTT solution was used after 48 h (Promega, USA)^84^. Using GraphPad Prism 7, as was previously reported in, we computed the survival rate in comparison to the control and obtained the IC_50_ values^85^.

### Topo II and DNA intercalation assay

Topoisomerase II (TopoGEN, Inc., Columbus) and DNA intercalator (methyl green (20 mg) and Calfthymus DNA (10 mg) were tested for their capacity to block the enzyme’s activity. Following equation was used to determine compound inhibition percentages: $100-[\frac{A\;\mathrm{control}}{A\;\mathrm{treated}}-\mathrm{Control})],$ IC_50_ was determined using GraphPad prism7 using inhibition curves at five different concentrations of each compound^86^.

## Investigation of apoptosis

### Annexin V/PI staining and cell cycle analysis

After incubation overnight, 6-well culture plates were populated with HepG2 cells (3–5 10^5^ cells/well). After that, compound 6 was used to treat the cells for 48 h at concentrations that were found to be IC50 for them. Cells and medium supernatants were then collected and washed in ice-cold PBS. The next step was suspending the cells in 100 µL of annexin binding buffer solution "25 mM CaCl2, 1.4 M NaCl, and 0.1 M Hepes/NaOH, pH 7.4” and incubation with “Annexin V-FITC solution (1:100) and propidium iodide (PI)” at a concentration equals 10 µg/mL in the dark for 30 min. The Cytoflex FACS system was then used to acquire the stained cells. cytExpert was used for the statistical analysis^87–89^.

### Real time-polymerase chain reaction for the selected genes

The gene expression of P53, Bax, Caspasses-3,8,9 as proapoptotic genes and Bcl-2 as an anti-apoptotic gene were evaluated to investigate the apoptotic pathway. The IC50 values for compound 6 were then used to treat HepG2 cells for 48 h. Afterwards, the standard operating procedure called for an RT-PCR reaction to be performed. The Ct values were then used to determine the relative expression of each gene in each sample, normalised to the β-actin as house-keeping gene^90–92^.

### In silico studies

Chimaera-UCSF and AutoDock Vina were used for molecular modelling research on a Linux-based system. Binding sites within proteins were identified by measuring the dimensions of grid boxes surrounding the co-crystallised ligands; this entire process was performed utilising Maestro to build and optimise the structures of both proteins and compounds. After standard procedures, the Topo II (PDB= 3qx3) protein structures were docked against the chemicals under study using AutoDock Vina^84^^,^^93–96^. Protein and ligand structures were optimised with Vina to make them more energetically favourable. The results of the molecular docking were interpreted by the binding activities in terms of the binding energy and the interactions between the ligand and the receptor. Chimaera was then used to complete the visualisation.

The Supporting Information is available free of charge at ^13^C NMR, ^1^H NMR, and IR spectra of the new synthesised compounds (PDF).

## Supplementary Material

Supplemental Material
