# Supplementary material for: Design, synthesis, molecular modelling and antitumor evaluation of S-glucosylated rhodanines through topo II inhibition and DNA intercalation
Source: J Enzyme Inhib Med Chem. 2023 Jan 11;38(1):2163996. doi: 10.1080/14756366.2022.2163996 (PMC9848385; doi:10.1080/14756366.2022.2163996)
Supplement: Supplemental Material [file IENZ_A_2163996_SM8184.pdf]

Design, Synthesis, Molecular Modelling and Antitumor Evaluation of *S*-Glucosylated Rhodanines Through Topo II Inhibition and DNA Intercalation

Ahmed I. Khodair*, Fatimah M. Alzahrani, Mohamed K. Awad, Siham A. Al-Issa, [d] Ghaferah H. Al-Hazmi, and Mohamed S. Nafie

Supplementary File

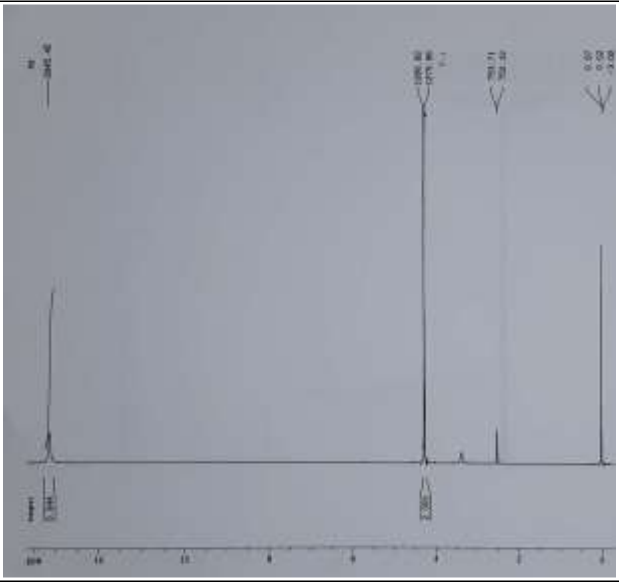	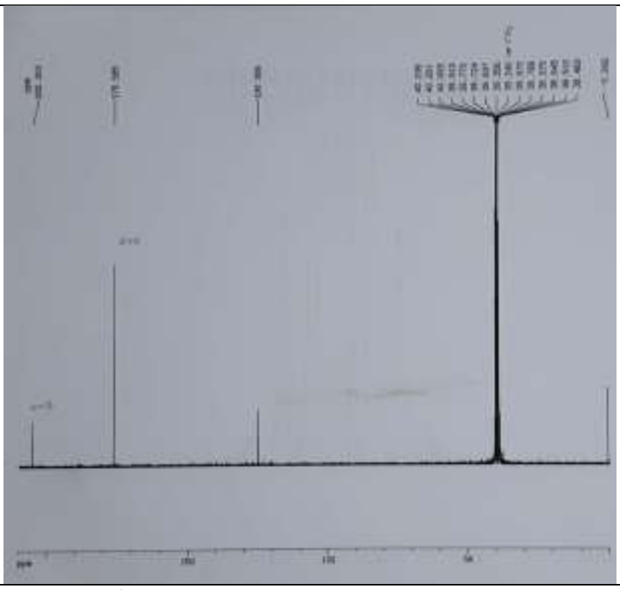
^{1}H-NMR spectrum of compound **1**	^{13}C-NMR spectrum of compound **1**
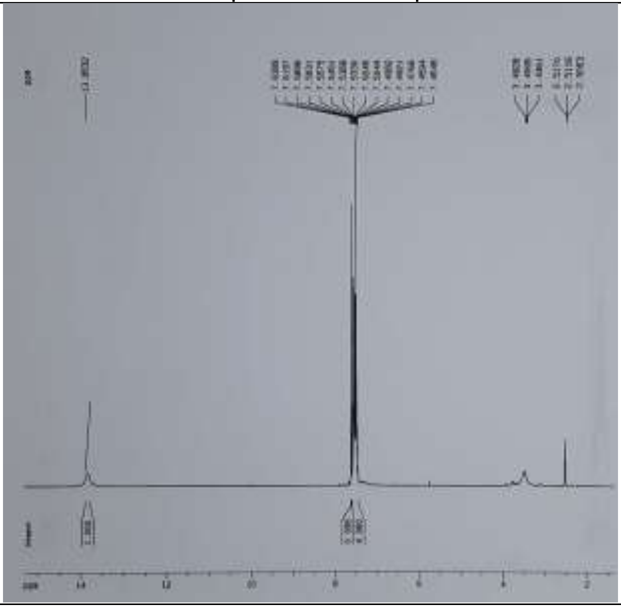	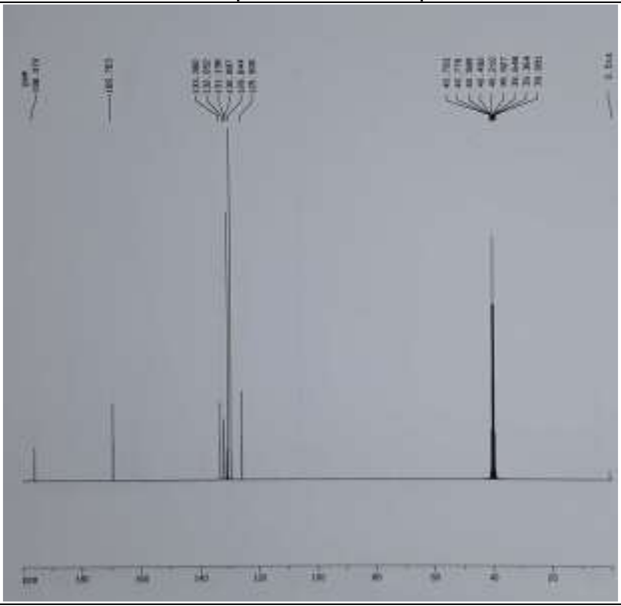
^{1}H-NMR spectrum of compound **3a**	^{13}C-NMR spectrum of compound **3b**

Information Classification: General

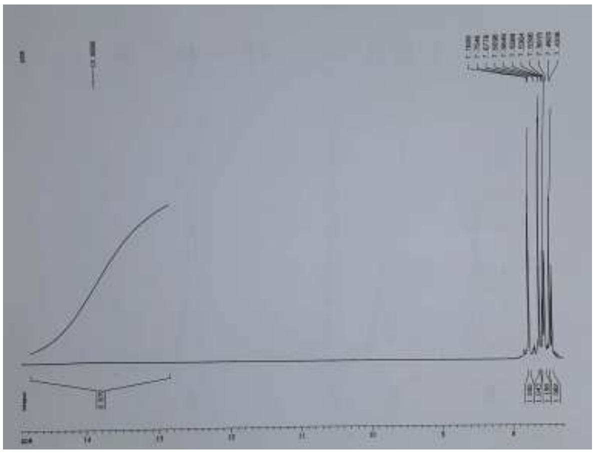	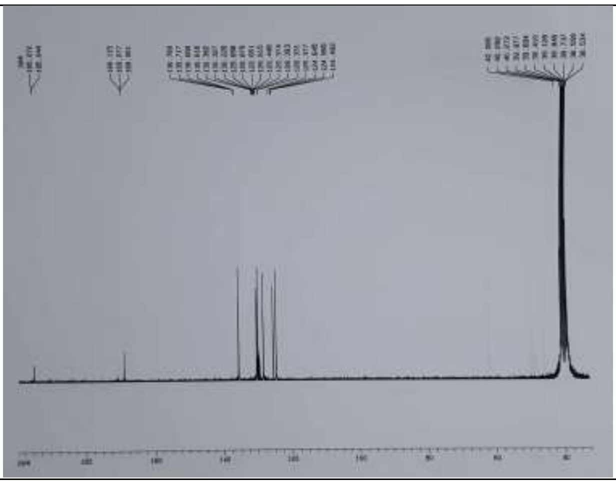
^{1}H-NMR spectrum of compound **3b**	^{13}C-NMR spectrum of compound **3b**
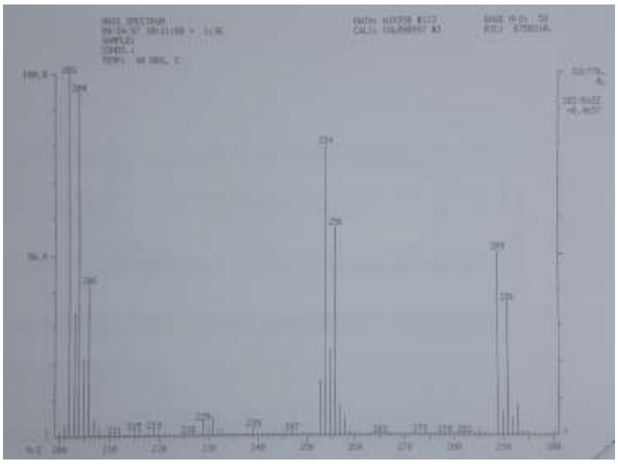	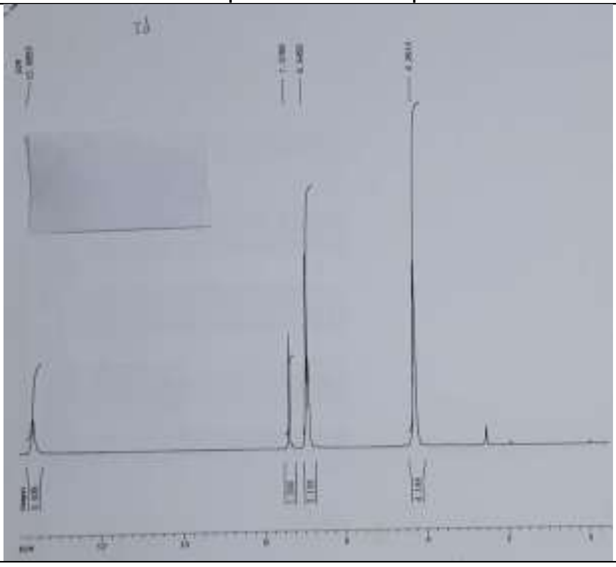
MS spectrum of compound **3b**	^{1}H-NMR spectrum of compound **3c**
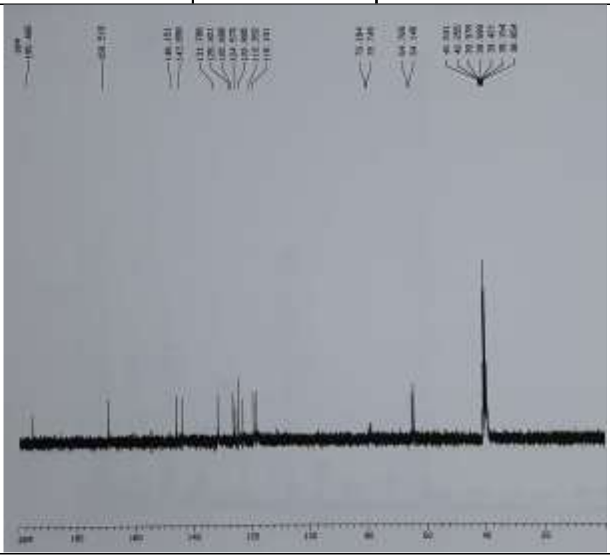	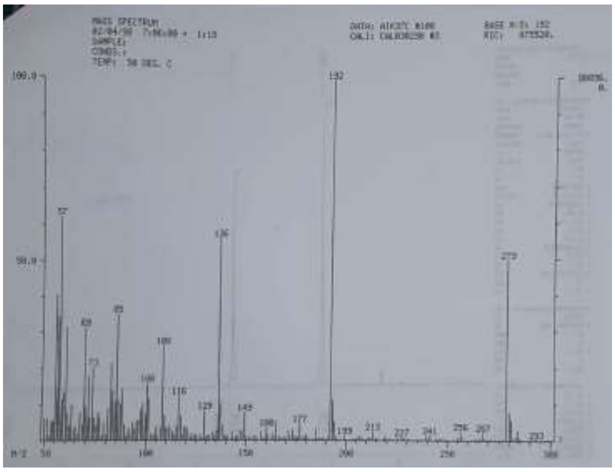
^{13}C-NMR spectrum of compound **3c**	MS spectrum of compound **3c**

Information Classification: General

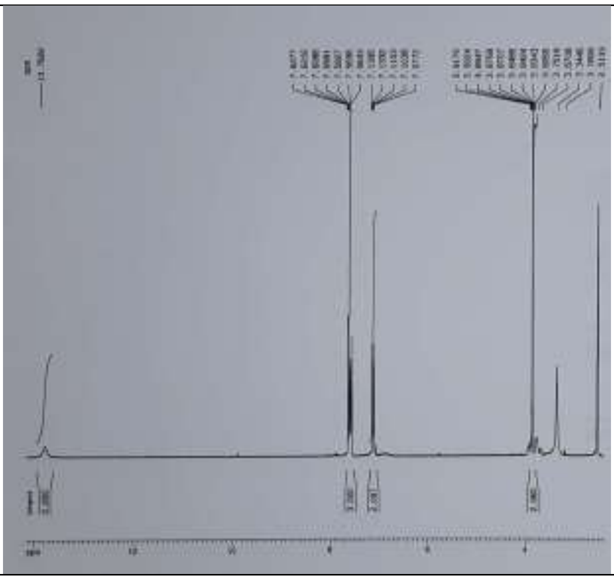	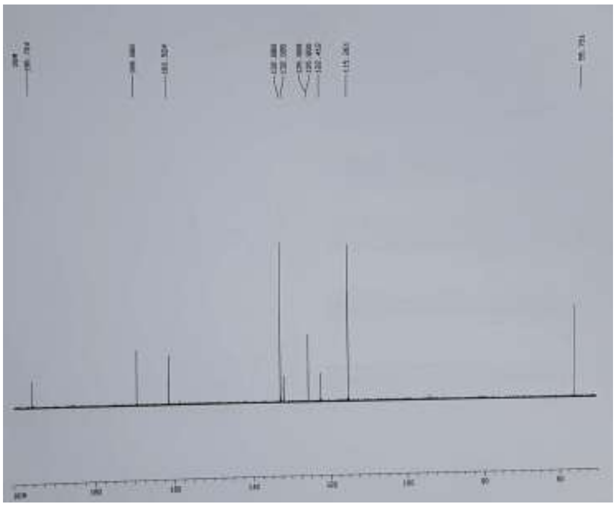
^{1}H-NMR spectrum of compound **3d**	^{13}C-NMR spectrum of compound **3d**
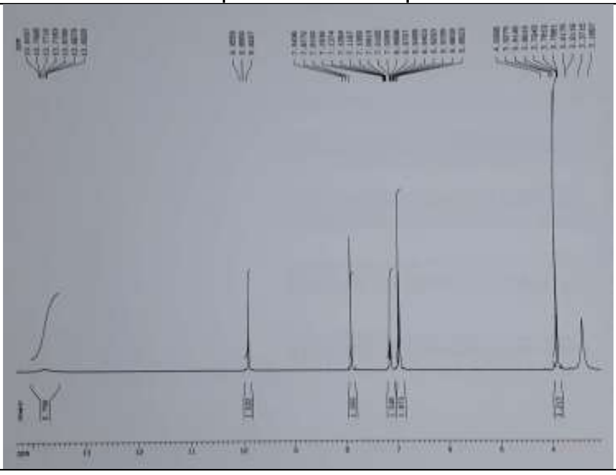	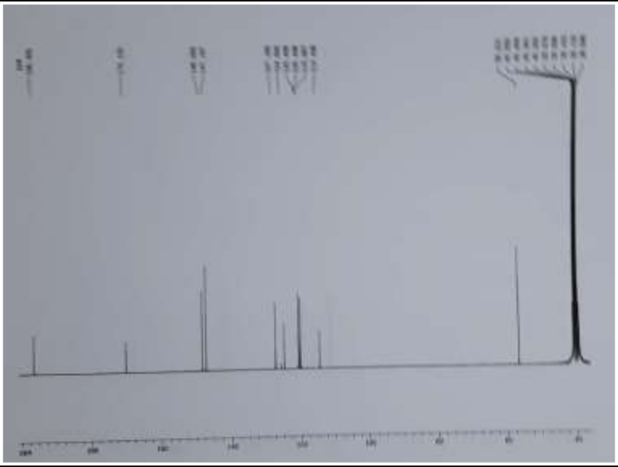
^{1}H-NMR spectrum of compound **3e**	^{13}C-NMR spectrum of compound **3e**
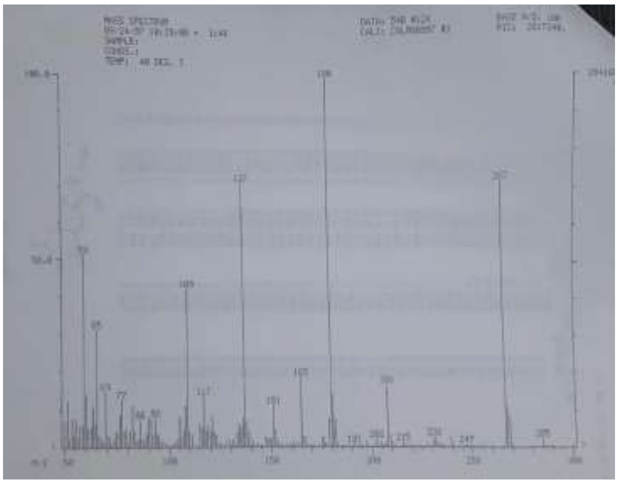	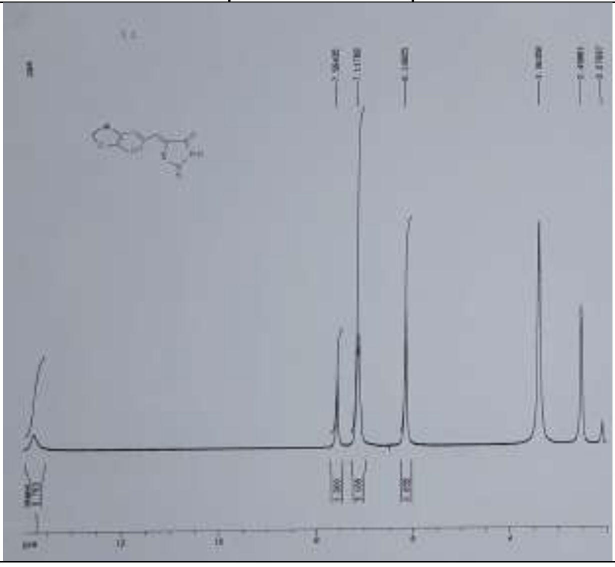
MS spectrum of compound **3e**	^{1}H-NMR spectrum of compound **3f**

Information Classification: General

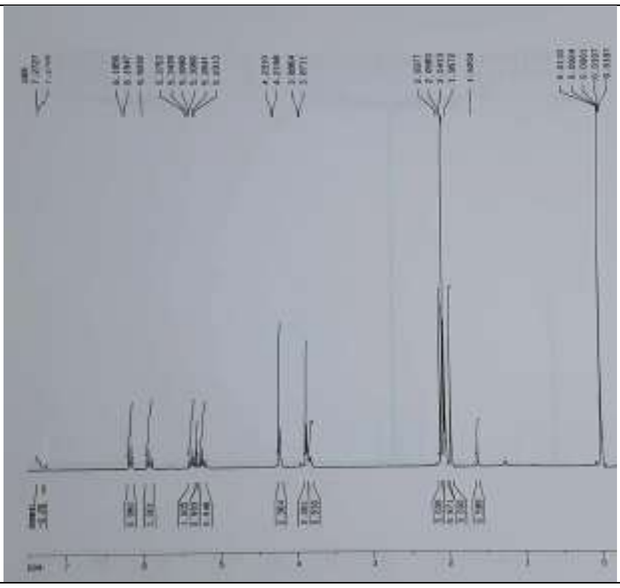

^{1}H-NMR spectrum of compound **6**

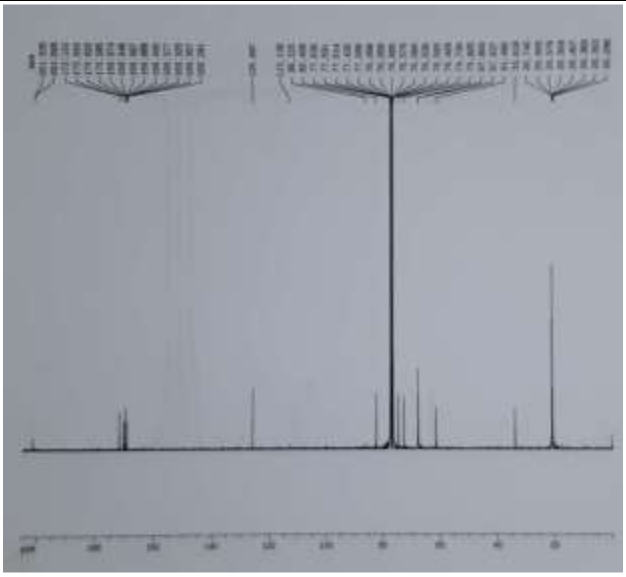

^{13}C-NMR spectrum of compound **6**

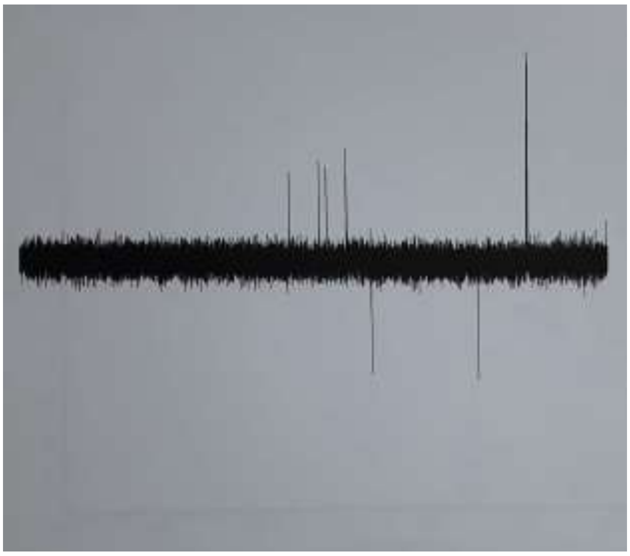

Dept135-NMR spectrum of compound **6**

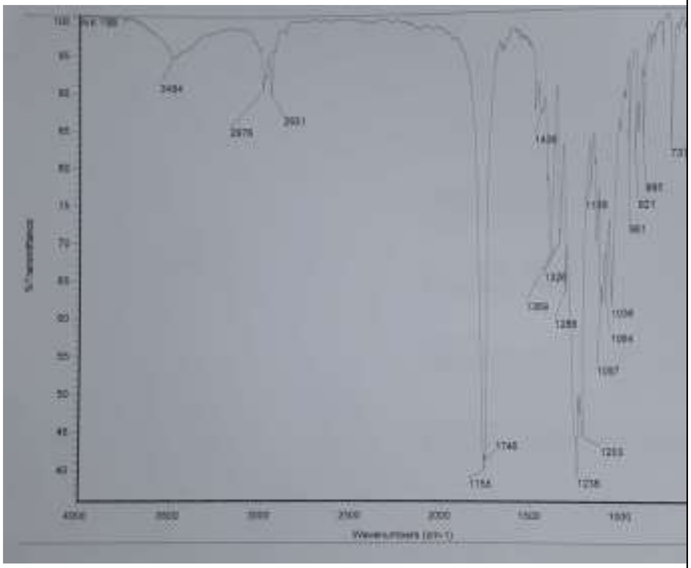

IR spectrum of compound **6**

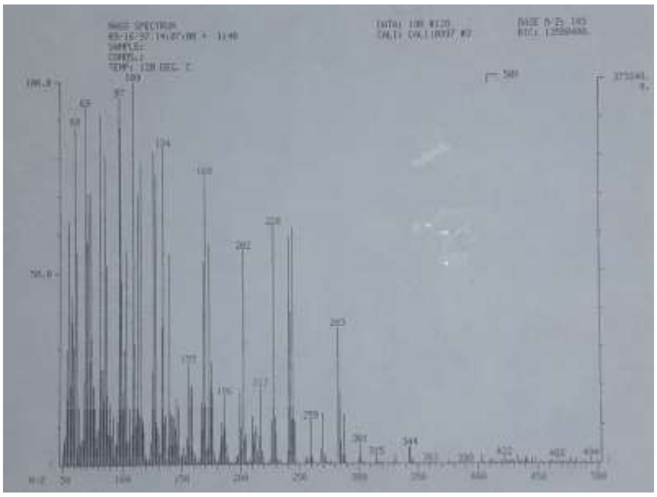

MS spectrum of compound **6**

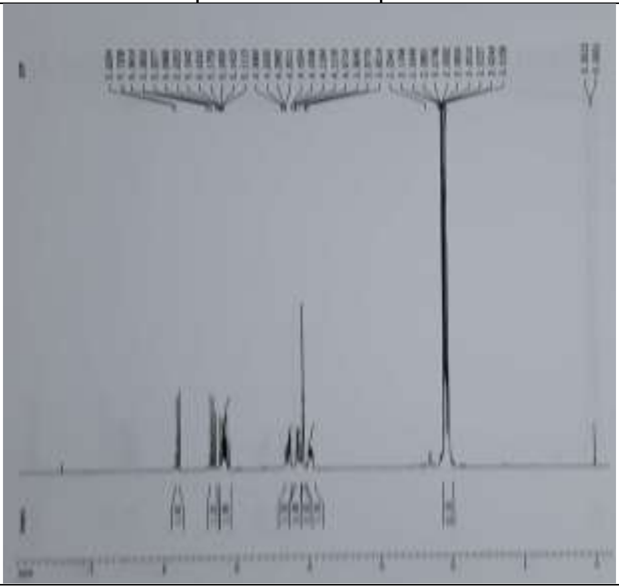

^{1}H-NMR spectrum of compound **7**

Information Classification: General

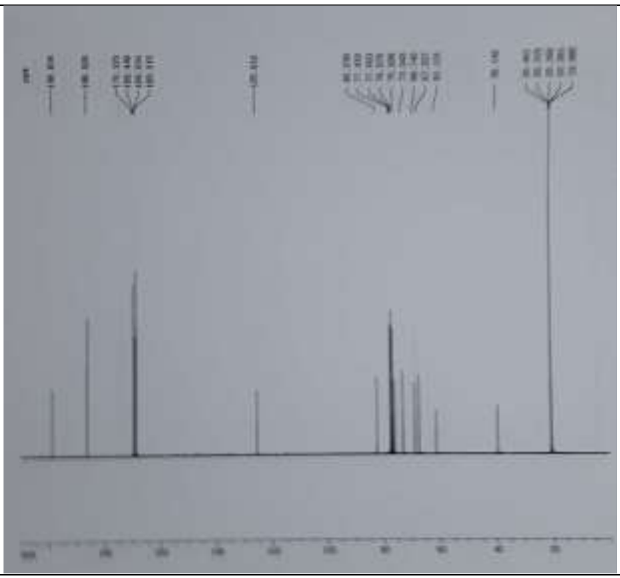	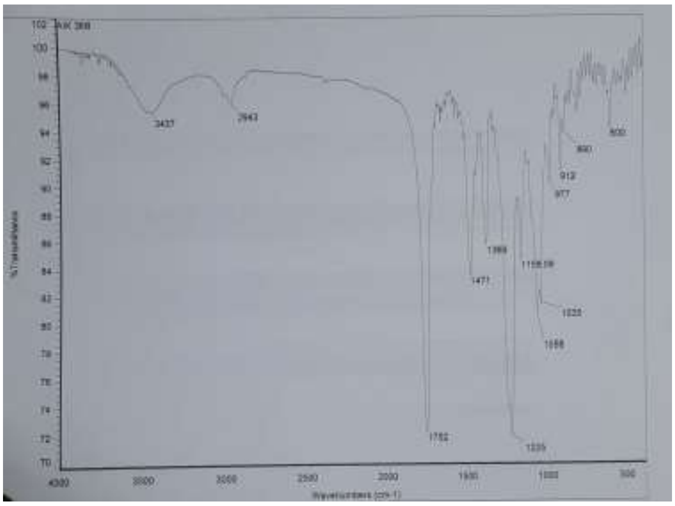
^{13}C-NMR spectrum of compound **7**	IR spectrum of compound **7**
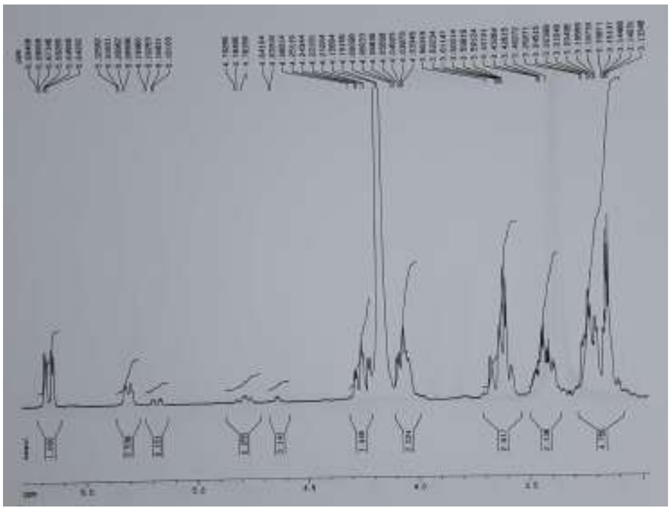	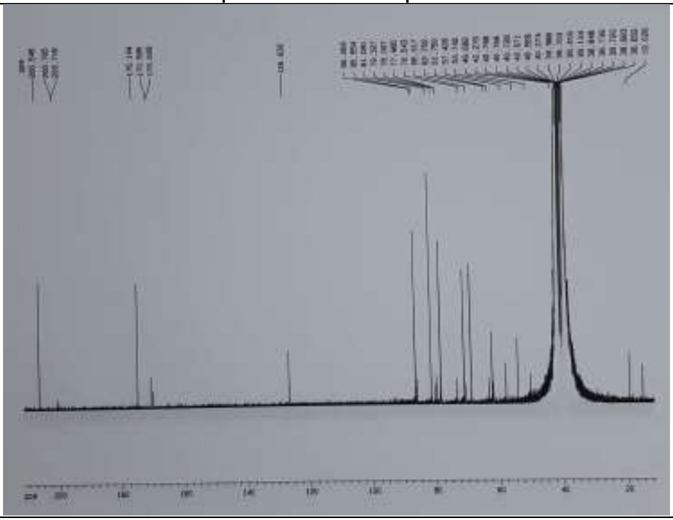
^{1}H-NMR spectrum of compound **8**	^{13}C-NMR spectrum of compound **8**
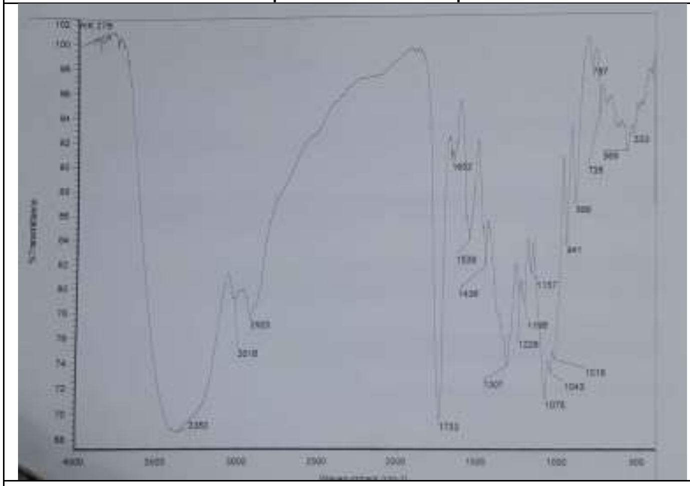	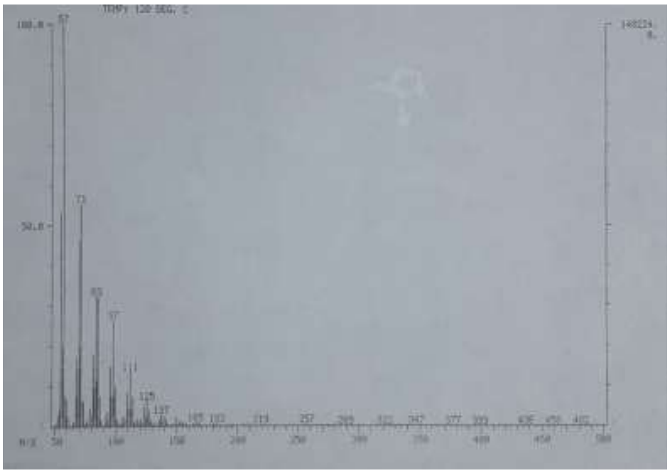
IR spectrum of compound **8**	MS spectrum of compound **8**

Information Classification: General

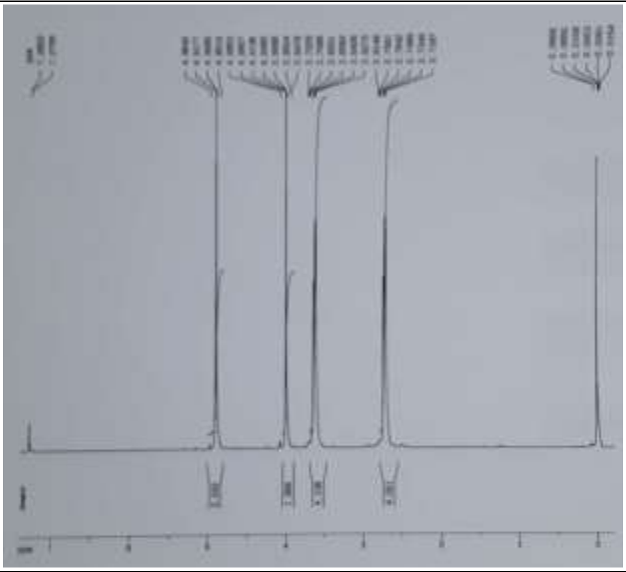	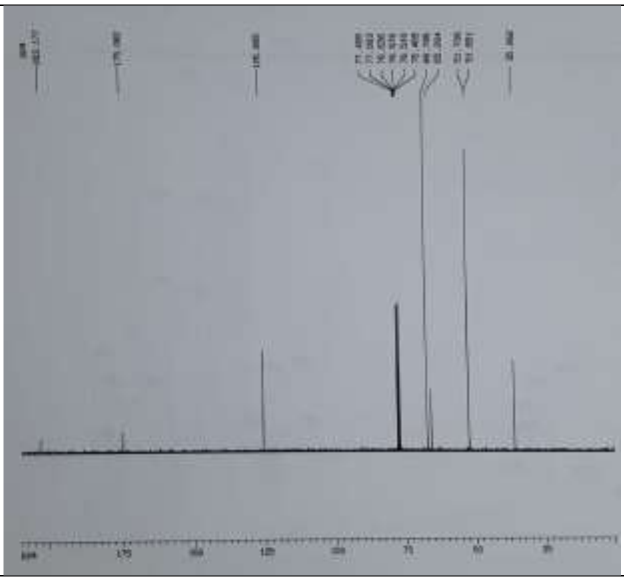
^{1}H-NMR spectrum of compound **10**	^{13}C-NMR spectrum of compound **10**
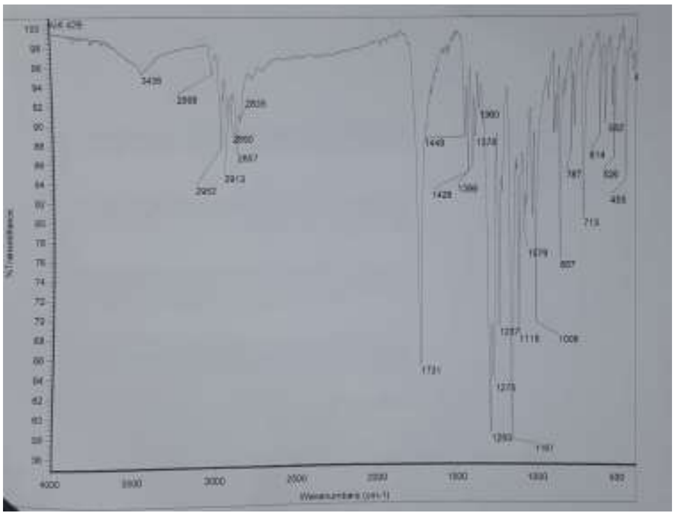	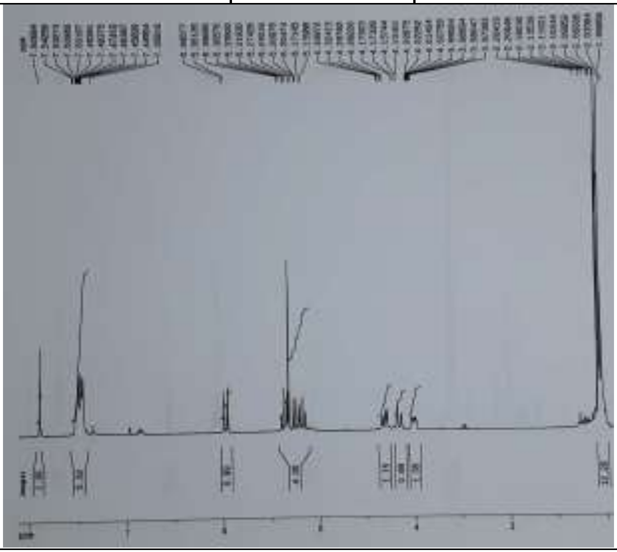
IR spectrum of compound **10**	^{1}H-NMR spectrum of compound **13a**
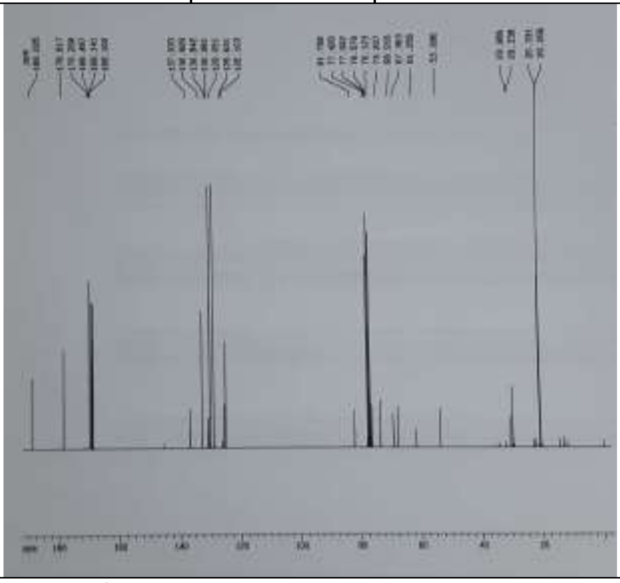	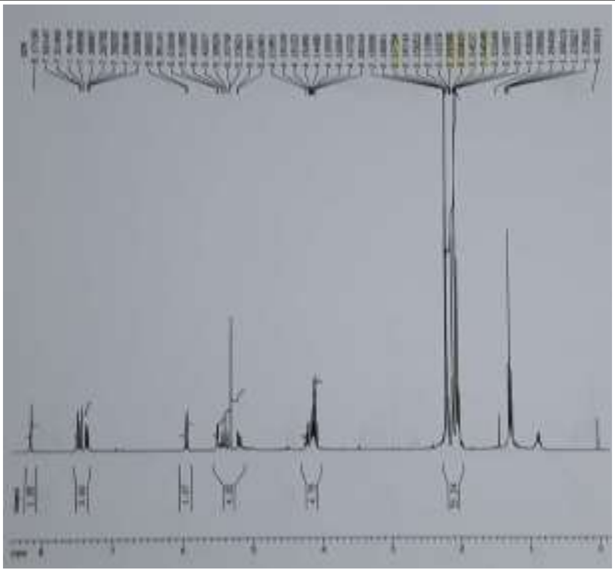
^{13}C-NMR spectrum of compound **13a**	^{1}H-NMR spectrum of compound **13b**

Information Classification: General

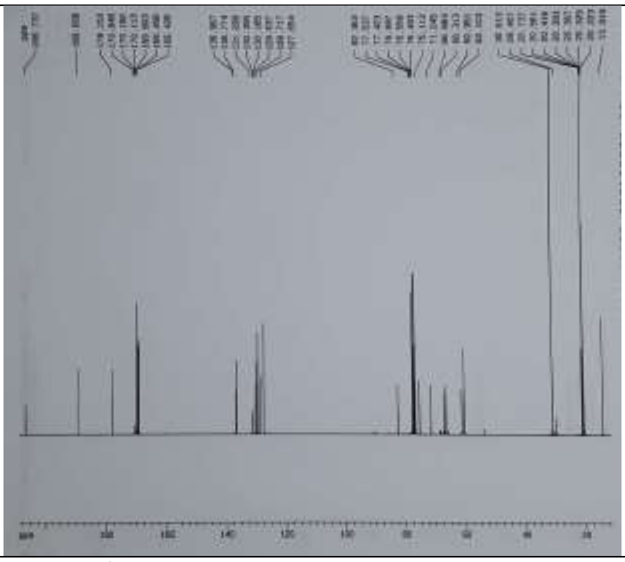	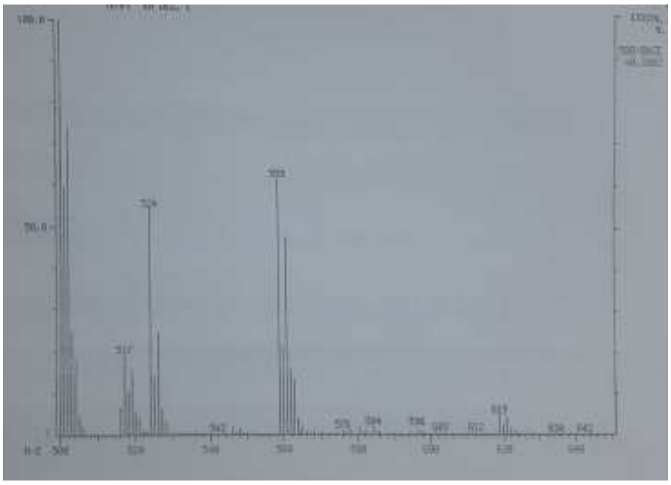
^{13}C-NMR spectrum of compound **13b**	MS spectrum of compound **13b**
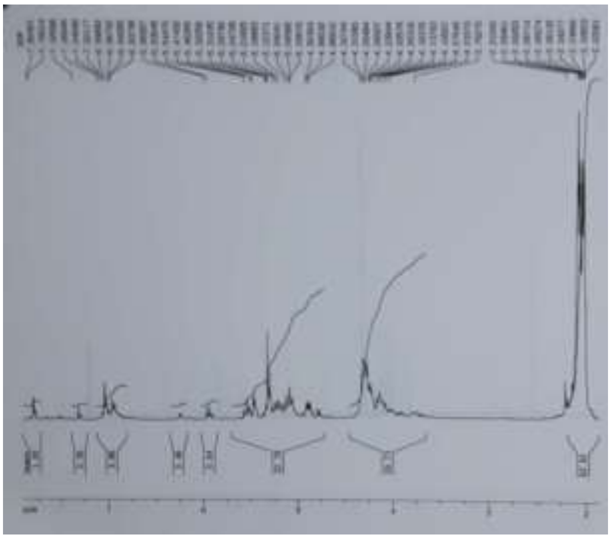	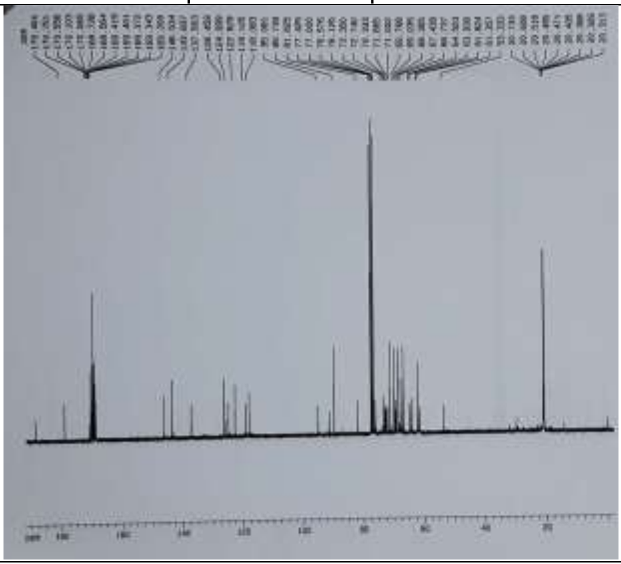
^{1}H-NMR spectrum of compound **13c**	^{13}C-NMR spectrum of compound **13c**
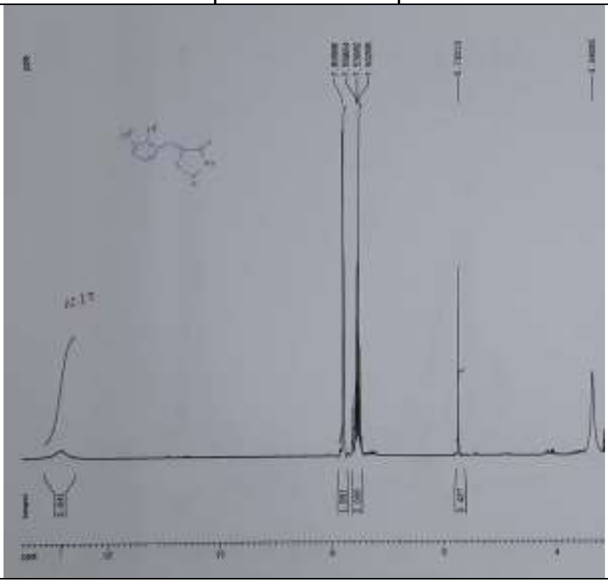	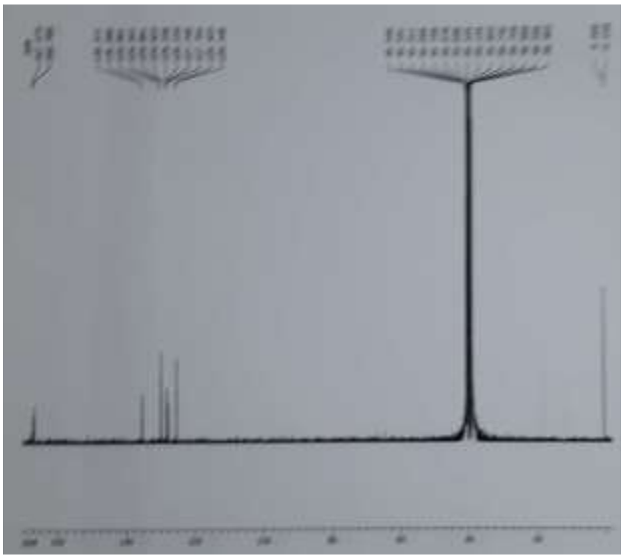
^{1}H-NMR spectrum of compound **14**	^{13}C-NMR spectrum of compound **14**

Information Classification: General
